# Cultivated sunflower ( *Helianthus annuus* L.) has lower tolerance of moderate drought stress than its con‐specific wild relative, but the underlying traits remain elusive

**DOI:** 10.1002/pld3.581

**Published:** 2024-04-04

**Authors:** Vivian H. Tran, Kristen M. Nolting, Lisa A. Donovan, Andries A. Temme

**Affiliations:** ^1^ Department of Plant Biology University of Georgia Athens Georgia USA; ^2^ Department of Plant Breeding Wageningen University & Research Wageningen Netherlands

**Keywords:** domestication, drought, *Helianthus annuus*, leaf theoretical maximum stomatal conductance, osmotic adjustment, resistance, stomatal density, stress, tolerance, vigor, wild relative

## Abstract

Cultivated crops are generally expected to have less abiotic stress tolerance than their wild relatives. However, this assumption is not well supported by empirical literature and may depend on the type of stress and how it is imposed, as well as the measure of tolerance being used. Here, we investigated whether wild and cultivated accessions of *Helianthus annuus* differed in stress tolerance assessed as proportional decline in biomass due to drought and whether wild and cultivated accessions differed in trait responses to drought and trait associations with tolerance. In a greenhouse study, *H. annuus* accessions in the two domestication classes (eight cultivated and eight wild accessions) received two treatments: a well‐watered control and a moderate drought implemented as a dry down followed by maintenance at a predetermined soil moisture level with automated irrigation. Treatments were imposed at the seedling stage, and plants were harvested after 2 weeks of treatment. The proportional biomass decline in response to drought was 24% for cultivated *H. annuus* accessions but was not significant for the wild accessions. Thus, using the metric of proportional biomass decline, the cultivated accessions had less drought tolerance. Among accessions, there was no tradeoff between drought tolerance and vigor assessed as biomass in the control treatment. In a multivariate analysis, wild and cultivated accessions did not differ from each other or in response to drought for a subset of morphological, physiological, and allocational traits. Analyzed individually, traits varied in response to drought in wild and/or cultivated accessions, including declines in specific leaf area, leaf theoretical maximum stomatal conductance (g_smax_), and stomatal pore length, but there was no treatment response for stomatal density, succulence, or the ability to osmotically adjust. Focusing on traits associations with tolerance, plasticity in g_smax_ was the most interesting because its association with tolerance differed by domestication class (although the effects were relatively weak) and thus might contribute to lower tolerance of cultivated sunflower. Our *H. annuus* results support the expectation that stress tolerance is lower in crops than wild relatives under some conditions. However, determining the key traits that underpin differences in moderate drought tolerance between wild and cultivated *H. annuus* remains elusive.

## 1 INTRODUCTION

As demand for food continues to increase with global population growth, crops today face numerous challenges that limit productivity (Foley et al., 2011). One proposed approach for reducing yield losses and improving environmental adaptation is to breed more stress tolerance into crops by incorporating traits from wild progenitors or relatives (Brozynska et al., 2016; Harlan, 1976; Tanksley & McCouch, 1997). This approach is based on the expectation that domestication has resulted in a loss of stress tolerance due to either a loss of genetic diversity or selection for vigor that led to the loss of traits expected to trade off with vigor, such as those important for defense and stress tolerance (Milla et al., 2015). However, there is limited support from empirical studies for demonstrating a loss of abiotic stress tolerance during domestication, especially under complex, multi‐faceted stresses like drought, which is an extremely pressing factor in crop productivity (FAO, 2017; Tardieu et al., 2018).

Plants respond to declining soil water availability at molecular, physiological, and morphological levels, resulting in lower performance assessed as growth and reproductive output, and there is a lot of variation among species and genotypes responses (Chaves et al., 2003; Seleiman et al., 2021; Tardieu et al., 2018). When considering specific traits thought to be related to overall “tolerance” of declining soil moisture, it can be useful to consider additional nomenclature that emphasizes the plant perspective of strategies (suites of plant traits) used to “resist” the external environmental drought, with strategies falling along a spectrum of being able to escape, avoid, and/or tolerate low plant water potentials (Delzon, 2015; Ludlow, 1989; Seleiman et al., 2021; Verslues et al., 2006). Escaping low plant water potential reflects adjustments in phenology to complete reproduction before water deficits affect performance. Avoiding low plant water potential reflects traits that allow a plant to prevent or postpone lower plant water status, generally by increasing water uptake capacity through modification of root traits and by reducing water loss through modification of water loss per unit leaf area and total leaf area relative to root uptake capacity. Tolerating low plant water potential reflects traits that allow plant tissue to survive more negative water potentials, including the ability to osmotically adjust to maintain turgor and water transport. In this manuscript, we will use the more commonly used term of “drought tolerance” as the general term for the overall performance response to the abiotic stress we are imposing, and discussing specific traits in the context of whether they contribute primarily to the ability to avoid or tolerate low plant water potentials. Promising targets for enhancing drought tolerance of crops include stomatal traits, osmotic adjustment, and altered biomass allocation (Tardieu et al., 2018; Killi et al. 2017; Dittberner et al., 2018; Bertolino et al., 2019; Haverroth et al., 2023; Karavolias et al., 2023).

Stomatal closure in response to water stress, reducing water loss and carbon gain and increasing water use efficiency, has long been studied (Fang & Xiong, 2015; Hetherington & Woodward, 2003; Huber et al., 2019; Xu & Zhou, 2008). More recently, the stomatal pore sizes and density of leaves produced under optimal and drought conditions have received attention as related to instantaneous rates of gas exchange and responses to drought (e.g., Bertolino et al., 2019; Dittberner et al., 2018; Doheny‐Adams et al., 2012; Drake et al., 2013; Hasanuzzaman et al., 2023; Karavolias et al., 2023; Milla et al., 2013; Xu & Zhou, 2008). The integrated effects of stomatal size and density can also be captured in the calculated trait of leaf theoretical (or anatomical) maximum stomatal conductance (g_smax_) (Cowan, 1977; Franks & Farquhar, 2001), which has shown promise for approximating instantaneous stomatal conductance measured under optimal water conditions (Dow et al., 2014; McElwain et al., 2016; Murray et al., 2020). Assessment of g_smax_ under non‐optimal conditions can also serve as an integrated proxy for stomatal density and size responses that reflect acclimation and adaptation to drought (Bertolino et al., 2019; Chatterjee et al., 2020). Stomatal trait responses, combined with reduced biomass allocation to leaves relative to roots, can contribute to the ability to avoid even lower plant water potentials.

Osmotic adjustment is the accumulation of osmotic compounds (compatible solutes) that do not interfere with normal metabolic functions. The osmotic compounds lower cell osmotic potential and allow plants to maintain turgor pressure, photosynthesis, metabolic activity, and growth (Blum, 2017; Cardoso et al., 2020; Jones & Turner, 1980; Turner, 2018). Because osmotic adjustment can help maintain metabolic activity and root growth under water deficits until water availability increases, it is a key trait to assess when investigating the potential for enhanced ability to tolerate low plant water potentials, though challenging to measure.

Sunflower (*Helianthus annuus* L.) is a model species that has been investigated for wild‐crop responses to abiotic stress (Koziol et al., 2012; Matesanz & Milla, 2018; Mayrose et al., 2011; Škorić, 2009; Tran et al., 2020). For drought, Mayrose et al. (2011) found that wild *H. annuus* were smaller than cultivated under control conditions and had greater drought tolerance based on a smaller proportional decline in plant height and longer survival in response to drought. Additionally, Koziol et al. (2012) found that wild *H. annuus* had higher drought tolerance than cultivated based on a smaller proportion of wilted leaves and stem wilting when aboveground biomass was included as a covariate. Inflorescence mass was smaller in wild *H. annuus* and had a smaller proportional decline in response to drought when compared to cultivated, supporting a potential tradeoff between growth and tolerance. However, Mayrose et al. (2011) and Koziol et al. (2012) both achieved drought by stopping watering and letting the potted plants deplete the soil, which may be a more drastic decline than experienced in most field conditions and can be confounded by plant size.

Here, we further explore the response of wild and cultivated *H. annuus* to drought using an automated irrigation system designed to maintain soil moisture treatments near predetermined setpoints for the whole soil volume (Nemali & van Iersel, 2006), minimizing the ability to avoid low plant water potentials through deeper rooting. This allowed for application of a more similar magnitude of stress applied across accessions despite differences in physiological responses and plant size. Additionally, we incorporated a larger number of both wild and cultivated accessions, used total plant biomass as a measure of growth, and explored more morphological and physiological traits potentially contributing to differences in tolerance. Objective 1 was to test whether wild and cultivated accessions differ in tolerance of moderate drought, with tolerance defined as the proportional reduction in biomass in response to drought. As part of supporting this metric of tolerance, we additionally assessed whether tolerance was associated with vigor (biomass in control treatment) because of previous observations of a general trade‐off between plant growth or size and stress tolerance in sunflowers under drought (Koziol et al., 2012; Mayrose et al., 2011) and salinity stress (Temme et al., 2019; Temme, Burns, & Donovan, 2020; Temme, Kerr, et al., 2020; Tran et al., 2020). Objective 2 was to test whether wild and cultivated accessions differ in response**s** of several traits expected to be associated with drought tolerance, including allocational, leaf morphological, stomatal, and physiological traits. Objective 3 was to test whether wild and cultivated *H. annuus* differ in trait associations with drought tolerance.

## 2 MATERIALS AND METHODS

### 2.1 Experimental design

*H. annuus* L. plants in two domestication classes (wild and cultivated) were grown in the University of Georgia Botany Greenhouses from July to August of 2019. Eight wild populations (hereafter “accessions”) were chosen from a geographically diverse spread across the range within the United States (Table S1). Eight inbred genotypes (hereafter “accessions”) of cultivated *H. annuus* (Table S1) were chosen to encompass a range of growth potentials expected to overlap with those of the wild accessions, based on previous phenotypic screens (McAssey et al., 2016; Temme et al., 2019; Temme, Burns, & Donovan, 2020; Temme, Kerr, et al., 2020; Tran et al., 2020).

Wild and cultivated *H. annuus* achenes (hereafter “seeds”) were germinated using different protocols to account for the lower germination rate and slower growth rate of wild *H. annuus*, resulting in seedlings at a similar developmental stage at treatment initiation. On July 1, 2019, wild *H. annuus* seeds were scarified by removing the blunt end of each seed and then placed on filter paper, moistened with a .45 g/L solution of Banrot fungicide (Everris NA112 Inc., Dublin, OH). Cultivated *H. annuus* seeds were started 8 days later on July 9, 2019, by sowing intact seeds in seedling trays filled with a soil substrate of a 3:1 ratio sand to calcinated clay (Turface Athletics, PROFILE Products, LLC, Buffalo Grove, IL). Post germination and expansion of cotyledons, all wild and cultivated seedlings were transplanted into individual 7.5 L pots (20 cm height, 26 cm diameter) filled with a 3:1 sand: Turface and supplemented with 60 g of 15‐9‐12 (N‐P‐K) Osmocote Plus blend with micronutrients (Osmocote, The Scotts Company), 15 ml of gypsum (Performance Minerals Corporation), and 15 ml of garden lime (Austinville Limestone).

### 2.2 Irrigation treatments

The eight wild and eight cultivated accessions were arranged in a split‐plot design, where each of six plots contained a subplot for a well‐watered control and a drought treatment, with one replicate of each accession placed randomly within each treatment subplot, for a total of 192 (2 treatments × 2 domestication classes × 8 accessions × 6 replicates) plants. An automatic irrigation system was used to continuously measure soil moisture and irrigate when soil moisture fell below the set soil moisture threshold values for each treatment (Nemali & van Iersel, 2006). The well‐watered control treatment had a set point of 35% soil moisture, which approximated field capacity. The drought treatment had a set point of 20% moisture that was expected to reduce growth but not cause terminal wilt for *H. annuus* in this soil substrate (Pilote, 2017).

Soil moisture was measured with soil moisture probes (EC‐5 probes, Meter Group, Pullman, WA) connected to a CR23X data logger (Campbell Scientific, Logan, UT) via two AM416 multiplexers (Campbell Scientific, Logan, UT). The 5 cm long probes were inserted into the soil so that they measured soil moisture from 2–7 cm depth out of approximately 16 cm total soil depth. For each accession by treatment combination, three of the six replicate pots had a soil moisture probe, and the soil moisture readings were averaged for these three pots. When the average soil moisture dropped below the applicable treatment set point (35% or 20%) for an accession, a signal from a relay driver (SDM‐CD16/AC‐16 Channel AC/DC Controller, Campbell Scientific, Logan, UT) was sent to a solenoid valve to open, which triggered a 30 s irrigation to each of the six replicate pots for that domestication class treatment combination. The 16 accessions and 2 treatments required 32 separate irrigation lines. The control versus drought treatments were imposed July 29 to August 12 (Figure S1).

### 2.3 Measurements

At the end of the 2‐week treatment period, each plant was measured for height (meter stick), basal stem diameter (digital calipers) 2 cm above soil surface, and Chlorophyll Index (Apogee MC‐100) of the Most Recently Fully Expanded Leaves (MRFEL) and then harvested. Biomass was separated into the following components: the pair of MRFELs, remaining leaves, stem (including developing bud, if present), and roots. The MRFEL pair was used for measurements made on fresh leaves at the time of harvest (see below), dried, and then combined with the remaining leaf biomass. The roots were washed free from soil particles prior to drying. All tissues were oven‐dried at 60°C for 72 h and weighed. Total biomass was calculated as the sum of all tissues per individual. For each individual plant, tissue mass fractions were calculated by dividing leaf, stem, and root biomass by the total biomass to get the leaf mass fraction (LMF), stem mass fraction (SMF), and root mass fraction (RMF) values, respectively.

At harvest, the two MRFELs from each individual were used for different measurements. One MRFEL was used to assess specific leaf area (SLA), succulence, and stomatal measurements. This MRFEL was weighed for fresh weight, scanned as a digital image with 300 dpi resolution for area with ImageJ (NIH, United States, http://rsb.info.nih.gov/ij/) (Schneider et al., 2012). The leaf blade left of the midrib was then excised and halved to create subsamples for making top and bottom leaf impressions. Each piece was pressed into dental putty (Defend VPS Putty, Mydent International Inc., Algonquin, IL) and given approximately 10 min to dry. After the putty had set, the leaf blade was peeled from the newly created putty mold and added to the remaining MRFEL tissue to dry. SLA was calculated as leaf area/dry weight (mm^2^/g). Leaf succulence was calculated as (leaf fresh weight − leaf dry weight)/area (g_water_ mm^−2^).

An epidermal impression was made from each putty mold by using clear nail polish to create a negative impression and affixing the impression to a microscope slide via clear tape (Gitz & Baker, 2009). A digital image (ZEN Digital Imaging for Light Microscopy, Zeiss, Oberkochen, DE) was taken of each leaf abaxial and adaxial epidermal impression. Subsequently, ImageJ was used to determine stomatal pore cell length and guard cell width as an average from eight representative stomata captured at random from the impression. Stomatal density was determined by counting the number of stomata in the area (magnified at 20X) and averaged per individual from four random areas of the impression, counting partial stomata along two edges of the frame. Leaf theoretical maximum stomatal conductance (g_smax_, mmol m^−2^ s^−1^) was calculated for each side by inputting the morphological stomatal measurements and stomatal density into a diffusion equation (developed from Cowan, 1977; Franks & Farquhar, 2001; see Dow et al., 2014, for equation) and summed as g_smax_ across leaf sides (Dow et al., 2014). Additionally, leaf gas exchange measurements were attempted with a LiCor 6400 just prior to the harvest, but equipment malfunction prevented the collection of usable data.

Contrasting with the morphological and stomatal measurements, the other MRFEL was used to assess osmotic potential and calculate osmotic adjustment using a method modified from (Bartlett et al., 2012). This MRFEL was initially bagged and kept in a cooler to prevent water loss and then rehydrated by inserting the petiole in deionized water in the dark for 24 h to achieve 100% relative water content (RWC). After leaf rehydration, a 6 mm diameter disk was punched out of the leaf blade in an area approximately 2.5 cm to the right of midvein leaf the base. The leaf disk was quickly cooled to −80°C in a freezer and then thawed to rupture cell walls. The remainder of the leaf was oven dried and weighed. A vapor pressure osmometer (Wescor Vapro 5520, Wescor Inc., Logan, UT) was used to measure the osmotic potential (MPa) of the cell sap from the leaf disks. Osmotic adjustment was calculated as the absolute difference between the mean stressed and mean control osmotic potential values for each accession paired by subplot.

### 2.4 Statistical analysis

All statistics were conducted using R (version 4.1.2) in Rstudio (version 2022.07.01), and all figures were made using package ggplot2 (Wickham, 2009). To obtain genotype means for all traits from our split plot design, a mixed effects model was fitted using the package lme4 (Bates et al., 2015) with accession and treatment and their interactions as fixed effects and drought treatment within plot as random factor. Trait means for each accession and treatment combination are available in Table S2.

To test for the effect of domestication class, drought treatment and their interaction we fitted an additional mixed effects model with domestication class, treatment, and their interaction as fixed effects and treatment within block and accession within domestication class as random factors. Significance of domestication class, drought treatment, and their interaction was tested using Wald's Chi‐square test on the mixed model (Fox & Weisberg, 2011). For each trait, differences among treatments and domestication classes were tested using Tukey's HSD (*p* = .05) corrected for multiple comparisons.

To quantify the degree of plasticity between treatments, we took the difference in natural log transformed trait value in drought minus log transformed trait value in control for each accession (following Temme, Kerr et al., 2020). This proportional (can be converted to Δ% vs. control using the equation Δ% = e^Δln[trait]^ − 1) metric of plasticity has the benefit of being viewpoint agnostic, the sign of the natural log difference changes when it is viewed from the control versus drought treatment. Moreover, a halving or doubling in trait value has the same magnitude of plasticity.

In quantifying an accession's tolerance, we took as a metric its proportional decline in biomass. This was calculated as ln (biomass drought)‐ln (biomass control), which can be converted to Δ% versus control using the equation Δ% = e^Δln(biomass)^ − 1. Those accessions that had a less negative proportional decline were viewed as having higher tolerance. We additionally assessed whether this measure of tolerance was related to vigor (defined as biomass in control treatment) with a linear model fitting tolerance (as proportional reduction in biomass) to vigor with domestication class as a covariate. Subsequently, we explored relationships of selected traits to tolerance within domestication class by a simple linear fit.

In addition to testing individual traits (above) for the effect of domestication class and drought treatment, a principal component analysis (PCA) was used to assess variation for a subset of seven morphological, physiological, and allocational traits (SLA, succulence, chlorophyll content, g_smax,_ LMF, RMF, SMF). Variation between domestication classes and treatments was tested using Hotellings‐T test on the first two principal components. Osmotic potential was excluded from the principal component analyses because it was available for only 13 of the 16 accessions and is analyzed separately as osmotic adjustment.

The relationship of tolerance to traits was assessed for multivariate traits and individual traits. For this analysis, the variation in the subset of seven morphological, physiological, and allocational traits was summarized using PCA for trait values in each treatment, control, and drought and for the plasticity in traits. The resultant major axes of variation (PC1 and PC2) in control, drought, and plasticity were then used in subsequent analyses. Separately for control, drought treatment, or their plasticity to tolerance, the relationship of each multivariate trait (PC1 and PC2) and individual trait was tested in a linear model fitting tolerance (as proportional reduction in biomass) to trait with domestication class as a covariate. Subsequently, we explored relationships of selected traits to tolerance within domestication class by a simple linear fit.

## 3 RESULTS

### 3.1 Do wild and cultivated accessions differ in drought tolerance??

**FIGURE 1:**
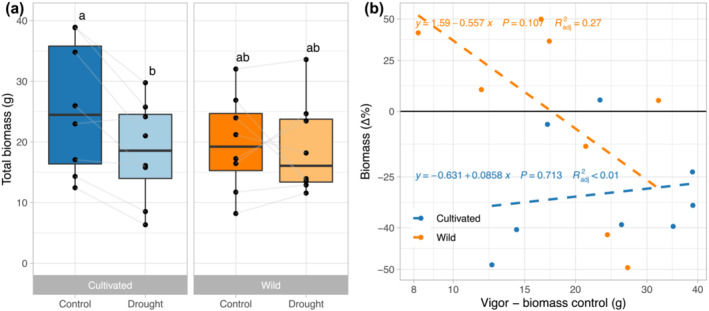
The effect of drought on biomass in wild and cultivated *H. annuus* . For each “box and whisker” plot, the dots reflect accession means, the box reflects the median (horizontal line) and quartiles, the whiskers represent the range of accession means excluding potential outliers, and dots outside of the whiskers represent potential outliers. (a) Total plant biomass by domestication class and treatments. Different lowercase letters represent significant differences (*p* < .05) based on post‐hoc contrasts from Tukey‐HSD tests. (b) The relationships between vigor ln (biomass under control conditions) and the proportional decline (Δ%) in biomass (assessed here as ln (biomass_droughtl_)‐ln (biomass_control_) and converted to Δ% = e^Δln(biomass)^ − 1) for wild and cultivated accessions in response to drought (ANOVA results: vigor *p* = .23, domestication class *p* = .07, interaction *p* = .11). Blue and orange dots indicate cultivated (*n* = 8) and wild accessions (*n* = 8), respectively; lines and equations show within domestication class results via linear regression. Dashed lines denote no significance at *P* < .05.

The drought treatment significantly decreased total biomass of cultivated *H. annuus* accessions by 24% (Table 1; Figure 1a). However, drought did not significantly decrease the biomass of the wild accessions (Figure 1a). Thus, the cultivated accessions had lower tolerance than the wild accessions, based on greater proportional reduction in biomass for cultivated accessions. The vigor (defined as biomass in control conditions) for the wild and cultivated accessions was largely overlapping in range and the means did not differ significantly (Table 1; Figure 1a). It is also interesting to note that the wild and cultivated biomass in the drought treatment did not differ significantly (Table 1; Figure 1a).

**TABLE 1:** Measured traits with their median value (based on accession means) and range (in parentheses) when grown under control and drought treatment, as well as an estimate of their plasticity between treatments.

	Cultivated							Wild									
Trait	Control			Drought			Plasticity (delta %)	Control			Drought			Plasticity	T	D	TxD
Total biomass (g)	24.48	a	(12.45–38.91)	18.56	b	(6.35–29.77)	−36.5 (−49–5.1)	19.22	ab	(8.2–32.03)	16.07	ab	(11.57–33.6)	7.4 (−49.62–49.83)	***	ns	**
Leaf mass (g)	1.88	a	(5.23–19.25)	9.72	b	(2.56–13.51)	−30.4 (−51.11–22.74)	9.48	ab	(3.91–16.52)	7.34	ab	(4.43–13.9)	−12 (−39.55–7.06)	**	ns	ns
Root mass (g)	4.44	a	(2.11–6.59)	2.75	b	(.85–4.95)	−30.9 (−59.87–.07)	2.78	ab	(1.1–5.01)	2.9	ab	(1.9–6.67)	31.4 (−48.74–180.33)	ns	ns	***
Stem mass (g)	7.61	a	(3.81–16.25)	5.81	b	(1.95–1.66)	−38.5 (−48.92–5.26)	6.33	ab	(2.71–9)	4.88	ab	(2.29–11.59)	−17 (−56.85–90.01)	***	ns	*
LMF (g_leaves_ g_plant_^−1^)	.42	a	(.39–.46)	.44	a	(0.4–.52)	3.2 (−6.25–21.43)	.48	a	(.36–.53)	.45	a	(.4–.47)	−9.9 (−13.27–18.23)	ns	ns	*
RMF (g_roots_ g_plant_^−1^)	.17	ab	(.12–.23)	.17	ab	(.12–.2)	−5.7 (−22.73–28.01)	.14	a	(.09–.24)	.2	b	(.14–.28)	44.7 (−4.03–168.69)	ns‐	ns	**
SMF (g_stem_ g_plant_^−1^)	.32	a	(.29–.42)	.31	a	(.27–.38)	−5.4 (−29.7–2.8)	.32	a	(.26–.36)	.29	a	(.22–.36)	−8 (−31.19–15.69)	*	ns	ns
SLA (m^2^ g^−1^)	436.67	ab	(236.19–552.29)	366.86	ab	(267.37–491.77)	−6.8 (−28.55–13.2)	429.96	a	(347.07–574.1)	345.03	b	(248.69–395.46)	−26.7 (−42.82 to −2.04)	***	ns	*
Succulence (g_water_ mm^−2^)	.03	a	(.02–.29)	.03	a	(.02–.04)	4.9 (−84.26–26.89)	.03	a	(.02–.03)	.03	a	(.02–.05)	−.6 (−22.22–69.27)	ns	ns	ns
Height (cm)	48.42	a	(35.92–7.42)	39.79	b	(24.08–56.5)	−20.5 (−32.95 to −1.89)	46.83	ab	(29.75–50.5)	39.12	ab	(26.3–60.42)	−8.2 (−27.72–22.05)	**	ns	***
Stem Diam. (mm)	16.64	a	(12.8–2.81)	13.44	bc	(9.2–15.82)	−22.2 (−31.96 to −7.09)	13.99	ab	(12.4–15.94)	11.56	c	(9.27–14.69)	−16.6 (−33.16 to −1.71)	***	*	**
Chlorophyl content (index)	20.08	a	(14.22–25.1)	27	b	(21.68–3.88)	33.5 (12.35–6.84)	19.73	a	(11.43–24.52)	25	b	(20.3–31.24)	20.4 (2.96–173.27)	***	ns	ns
Osmotic potential (MPa)	1.23	a	(.98–1.41)	1.52	a	(1.19–1.71)	24 (9.16–64.69)	1.31	a	(1.09–1.38)	1.46	a	(1.21–1.73)	6.5 (−2.75–58.49)	ns‐	ns	ns‐
g_s_max (mmol m^2^ s^−1^)	38.13	a	(29.69–59.5)	3.74	b	(21.65–35.88)	−18.3 (−39.73–4.16)	32.2	ab	(29.96–43.65)	28.27	ab	(23.89–46.12)	−6.6 (−24.89–25.73)	*	ns	ns‐
Abaxial stomatal density (mm^−2^)	27.9	a	(19.54–38.5)	23.9	a	(19.69–28.21)	−17.9 (−26.73–11.72)	26.21	a	(22.71–31.5)	26.96	a	(22.42–28.99)	−1.2 (−13.82–24.09)	ns	ns	*
Adaxial stomatal density (mm^−2^)	24.66	a	(17.04–33.45)	22.12	a	(16.78–24.32)	−9.4 (−39.6–16.14)	23.93	a	(2.83–28.45)	23.39	a	(2.16–28.42)	−2.2 (−29.15–18.65)	ns	ns	ns
Abaxial pore length (μm)	597.21	a	(509.72–636.41)	478.73	b	(441.88–567.96)	−12.6 (−25.47 to −6.4)	549.93	ab	(514.34–61.85)	509.96	b	(445.72–539.56)	−7.6 (−19.95 to −1.31)	***	ns	ns
Adaxial pore length (μm)	531.21	a	(46.84–631.48)	507.02	ab	(420.53–546.96)	−9.3 (−21.53–.55)	498.37	a	(447.73–572.39)	45.68	b	(331.12–531.42)	−14.3 (−32.64–12.87)	***	*	ns
Abaxial guard cell width (μm)	157.21	a	(136.95–173.97)	156.18	a	(116–163.24)	−2.8 (−3.72–15.12)	158.13	a	(14.07–166.55)	151.85	a	(14.87–166.36)	−2.7 (−7.52–7.83)	ns	ns	ns
Adaxial guard cell width (μm)	149.12	a	(137.72–154.77)	141.64	ab	(122.04–156.89)	−5.2 (−21.15–11.43)	137.92	ab	(127.38–157.75)	128.94	b	(89.01–157.93)	−6.5 (−39.66–13.31)	ns	**	ns

*Note*: Different lowercase letters represent significant (*p* < .05) Tukey's post hoc differences among domestication classes and treatments for each trait from the full treatment × domestication class model. Plasticity was calculated as the difference in natural log transformed values (ln (drought)‐ln (control)) but converted here to Δ% = (e^Δln[trait]^ − 1) for ease of interpretation for each of the accessions. Stars indicate significance (Wald's Chi‐square of treatment [T], domestication class [D], and their interaction [T × D]).

Abbreviations: LMF, leaf mass fraction; ns, non‐significant; RMF, root mass fraction; SLA, specific leaf area; SMF, stem mass fraction; succulence, chlorophyll content, and g_s max_, leaf theoretical maximum stomatal conductance.

* *P* < .05.

** *P* < .01.

*** *P* < .001.

Explicitly taking variation in vigor into account, we found no relationship between the proportional reduction in biomass in response to drought and vigor though there was a trend for vigor and an interaction between vigor and domestication class (Figure 1b, *p* = .23 for vigor, *p* = .07 for domestication class, and *p* = .11 for interaction). Within each domestication class, further linear regression analysis confirmed that there was no relationship between vigor and proportional reduction in biomass (Figure 1b). Thus, the effect of vigor was not taken into consideration for the subsequent analyses.

### 3.2 Do wild and cultivated accessions differ in trait responses to drought?

**FIGURE 2:**
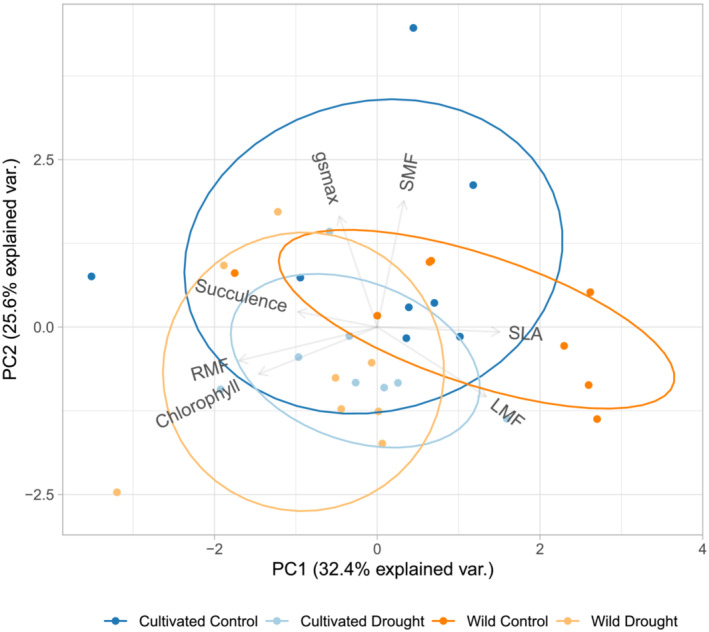
Principal component analysis (PCA) of a subset of seven morphological, physiological, and allocational traits for wild (*n* = 8) and cultivated (*n* = 8) *H. annuus* in control and drought treatments. Trait abbreviations: LMF, leaf mass fraction; RMF, root mass fraction; SMF, stem mass fraction; SLA, specific leaf area; succulence, chlorophyll content, and g_s max_, leaf theoretical maximum stomatal conductance; SD, stomatal density; PL, pore length; GCw, guard cell width, a proxy for pore depth. Due to missing osmotic potential data for several accessions, it was excluded from this PCA analysis. Blue and orange symbols indicate cultivated and wild accessions, respectively. Darker and lighter hues indicate control and drought treatment, respectively. Hottelings‐T^2^ test indicated no significant differences among the four treatment and domestication classes.

We first used a PCA to examine variation in a subset of seven morphological, physiological, and allocational traits: SLA, succulence, chlorophyll content, and g_smax_ (leaf theoretical maximum stomatal conductance); LMF, RMF, and SMF. Across both treatments and domestication classes, 58% of the variation in our traits was captured by the first two principal components (Figure 2). In this multivariate assessment, post hoc contrasts indicated there were no significant differences among the four treatment and domestication class combinations, although there was a trend for wild accessions in the control treatment to differ from cultivated accessions in the drought treatment (*p* = .08). Key traits contributing to PC1 were RMF and SLA. Key traits contributing to PC2 were SMF and g_smax_ (Table S3). In order to check the potential influence of g_smax_ component traits on this analysis, we ran an additional PCA that included the subset of seven morphological, physiological, and allocational traits, plus the six stomatal density and size traits used to calculate g_smax_ (Figure S2), but there were still no significant differences among the four treatment and domestication class combinations.

We further investigated the seven morphological, physiological, and allocation traits, as well as stomatal size and density, for effects of treatment and domestication class (Table 1). There was a significant treatment effect and/or interaction of treatment and domestication class (T and TxD columns in Table 1), indicating a treatment response in the wild and/or cultivated accessions, for many traits, including LMF, RMF, SMF, SLA, leaf succulence, leaf theoretical maximum stomatal conductance (g_smax_), abaxial stomatal density, abaxial stomatal pore length, and adaxial stomatal pore length. There was no treatment response in the wild and/or cultivated for succulence, adaxial stomatal density, abaxial guard cell width, and adaxial guard cell width.

**FIGURE 3:**
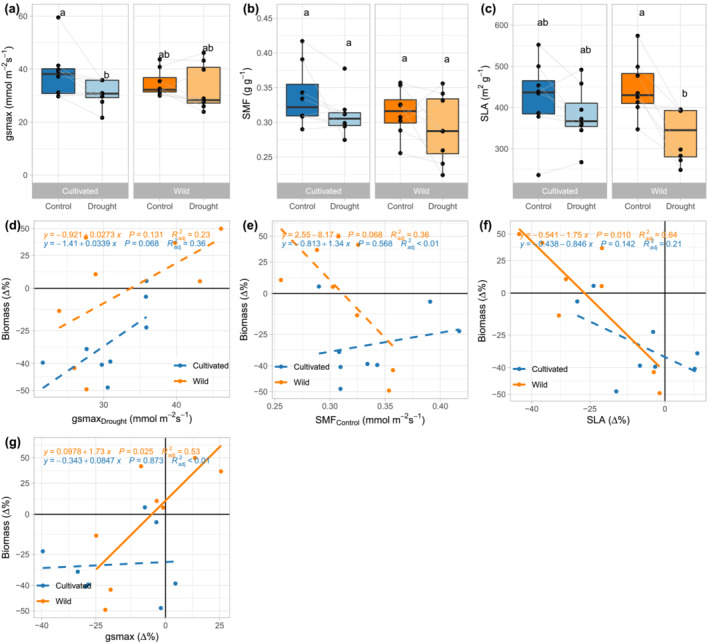
The effect of drought on g_s max_ (leaf theoretical maximum stomatal conductance), SMF (stem mass fraction), and SLA (specific leaf area) in wild (*n* = 8) and cultivated (*n* = 8) *H. annuus* . (a) g_s max_, (b) SMF, and (c) SLA by domestication class and treatment. For each “box and whisker” plot, the dots reflect accession means, the box reflects the median (horizontal line) and quartiles, the whiskers represent the range of accession means excluding potential outliers, and dots outside of the whiskers represent potential outliers. Different lowercase letters represent significant differences (*p* < .05) based on post hoc contrasts from Tukey‐HSD tests (Table 1). The relationships between (d) g_s max_ in drought, (e) SMF in control, (f) SLA plasticity, (g) g_s max_ plasticity, and Δ% (proportional decline in biomass, assessed here as ln (biomass_droughtl_)‐ln (biomass_control_), and converted to Δ% = e^Δln(biomass)^ − 1) for wild and cultivated accessions in response to drought. Blue and orange dots indicate cultivated and wild accessions, respectively. Lines and equations show within domestication class results via linear regression. Dashed lines denote no significance.

Focusing on SMF, SLA, and g_smax,_ for which among accession variation was subsequently found to be associated with drought tolerance (see the next section; Table 2), post hoc contrasts indicated differences among only a few of the means for the accession by domestication classes groupings (Table 1; Figure 3). For SMF, while there was a significant treatment effect, the within domestication classes decrease in SMF were non‐significant (Table 1; Figure 3b). For SLA, the wild accessions experienced a significant decrease in response to drought, whereas cultivated accessions did not (Table 1; Figure 3c). For g_smax_, cultivated accessions experienced a significant decrease in response to drought, whereas wild accessions did not (Table 1; Figure 3a). When considering stomatal traits used to calculate g_smax_, g_smax_ was influenced by both stomatal density and stomatal size (Table S4; Figure S3).

**TABLE 2:** Associations of drought tolerance (measured by proportional reduction of biomass; see Figure 1), with trait variation of multivariate traits (PC1 and PC2 scores from PCA of the seven morphological, physiological, and allocational traits, see Figure 3), and individual traits under control, drought, and the plasticity between them.

	Control			Drought			Plasticity		
Trait	P (trait)	P (Dom)	P (TxD)	P (trait)	P (Dom)	P (TxD)	P (trait)	P (Dom)	P (TxD)
PC1	.27	.15	.81	.23	.1	.58	.06	.31	1
PC2	.69	.07	.17	.29	.12	.45	.06	.17	.32
LMF (g_leaves_ g_plant_^−1^)	.69	.73	.82	.53	.12	.15	.89	.10	.32
RMF (g_roots_ g_plant_^−1^)	.97	.66	.38	.62	.68	.47	.60	.17	.72
SMF (g_stem_ g_plant_^−1^)	.13	**.03**	**.04**	.48	.82	.96	.71	.06	.39
SLA (m^2^ g^−1^)	.31	.49	.32	.07	.45	.60	**.003**	.59	.22
Succulence (g_water_ mm^−2^)	.48	.62	.48	.80	.70	.99	.90	.10	0.84
Chlorophyl content (index)	.45	.58	.78	.73	.87	.93	.75	.46	.87
g_s_max (mmol m^2^ s^−1^)	.62	.67	.92	**.03**	.56	.80	**.04**	**.03**	**.05**
Abaxial stomatal density (mm^−2^)	.76	.60	.79	.52	.63	.72	.85	.13	.75
Adaxial stomatal density (mm^−2^)	.83	.50	.66	.12	.88	.99	.46	.07	.36
Abaxial pore length (μm)	.25	.46	.38	.58	.74	.83	.86	.79	.16
Adaxial pore length (μm)	.88	.14	.18	.17	.95	.80	.77	**.03**	.24
Abaxial guard cell width (μm)	.64	.93	.86	.39	.50	.63	.61	.13	.72
Adaxial guard cell width (μm)	.40	.44	.38	.90	.48	.55	.18	.19	.11

*Note*: Values show *p* value significance of ANOVA model fit of the proportional reduction in biomass to trait with domestication class as a covariate.

*P* values <.05 highlighted in black.

**FIGURE 4:**
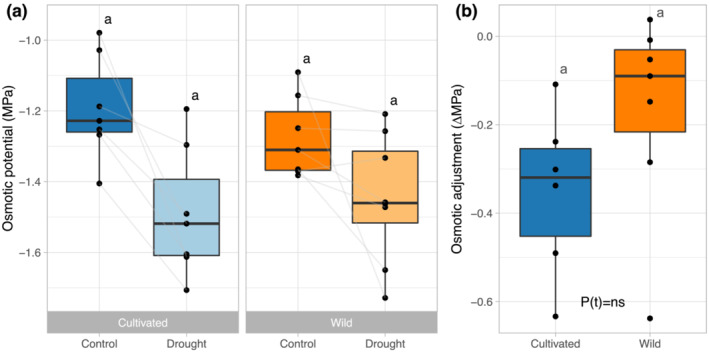
The effect of drought on osmotic characteristics of wild and cultivated sunflower. For each “box and whisker” plot, the dots reflect accession means, the box reflects the median (horizontal line) and quartiles, the whiskers represent the range of accession means excluding potential outliers, and dots outside of the whiskers represent potential outliers. (a) Osmotic potential of wild and cultivated accessions under control and drought treatment. Lowercase letters denote no significant differences (*p* < .05) based on post hoc contrasts from Tukey‐HSD tests. (b) Osmotic adjustment in response to drought in wild and cultivated *H. annuus* . Lowercase letters denote no significant difference between domestication classes (*p* < .05) based on T‐test result (note that removal of lowest wild outlier does result in significant, *p* < .02, differences).

Osmotic adjustment, calculated as the difference between osmotic potential of control and drought plants of each accession, did not differ between wild and cultivated accessions (Figure 4, *p* = .15). For the sake of completeness, we did explore the effect of one wild accession as a potential outlier. While removing the wild accession with the highest osmotic adjustment as an outlier did result in detection of significantly greater osmotic adjustment in cultivated than wild accessions (*p* < .02), there was no biological justification for removing it (e.g., accession replicates were consistent in value, and there were no plant health or methodological red flags). Additionally, the inclusion or exclusion of that accession did not change the results of our subsequent analysis of trait associations with tolerance (see the next section), so we proceeded with all available accession data included.

### 3.3 Do wild and cultivated accessions differ in traits associated with tolerance?

We assessed whether drought tolerance measured as a proportional reduction in biomass was associated with the trait variation among accessions and whether this association differed by domestication class. We first summarized variation in the subset of seven morphological, physiological, and allocational traits with three separate PCAs: (1) traits in the control treatment, (2) traits in the drought treatment, and (3) trait responses to the treatments or plasticity between treatments (Figure S4). Drought tolerance was not associated with trait variation captured by any of the principal component scores (P [trait] column in Table 2), although there was a trend for plasticity in PC1 and PC2 to explain tolerance. However, this trend did not differ by domestication class (P [Dom.] Table 2).

When the individual traits were assessed, three showed associations with tolerance: g_smax_ in the drought treatment, SMF in the control treatment, SLA plasticity, and g_smax_ plasticity (Table 2 (P [trait] column), Figure 3d–g, respectively). However, g_smax_ plasticity was the only trait that was associated with tolerance and differed by domestication class, and the relationship was relatively weak (Table 2). Wild accessions with a greater decline in g_smax_ in response to drought also had a greater decline in biomass and thus had less tolerance (Figure 3g). When individual stomatal density and stomatal size traits were assessed, none showed associations with tolerance (Table 2).

Osmotic potential was analyzed separately due to missing data for several accessions. There was no relationship between the extent of osmotic adjustment and tolerance (*p* < .23) nor an interaction between domestication classes (*p* < .57).

## 4 DISCUSSION

### 4.1 Wild and cultivated *H. annuus* differ in their performance in response to drought

We found that cultivated accessions had a larger proportional effect of drought stress, here measured as biomass reduction, providing further support for the expectation that cultivated accessions of *H. annuus* have less drought tolerance than wild accessions (Ashraf, 2004; Koziol et al., 2012; Mayrose et al., 2011; Nayyar et al., 2005). However, the wild and cultivated accessions did not differ for biomass in the drought treatment, which may be on interest for investigators more interested in that alternative measure of tolerance. Because responses to drought are highly complex, several open questions remain, including whether differences between wild and cultivated responses to drought will hold under different duration and intensities of drought, life stages, and opportunities for recovery if the drought ceases.

Surprisingly, we did not observe a trade‐off between vigor (defined as biomass in control conditions) and the proportional decline in biomass. This was unexpected given that prior work on sunflower has shown this negative relationship under salinity stress: plants with more biomass in control treatment had a larger proportional decline in response to salinity stress (Temme et al., 2019; Temme, Burns, & Donovan, 2020; Temme, Kerr, et al., 2020; Tran et al., 2020). The differences between the salinity stress and drought stress effects may lie at the heart of this (Forni et al., 2017; Munns, 2002). For example, during salinity stress, high vigor can serve to dilute excess toxic ions, but this plays no role during lack of water (Yeo & Flowers, 1986). Thus, our results highlight that even within one species, the role of vigor in stress responses is difficult to generalize across different abiotic stresses, and variation in how a given stress is perceived/experienced.

### 4.2 Multivariate trait suites are similar across treatments and domestication classes, but some individual trait responses are promising

In multivariate analysis, the subset of morphological, physiological, and allocational traits was comparable for wild and cultivated accessions in each treatment, with only trends for differences. This was surprising given the greater biomass reduction in biomass for cultivated as compared to wild accessions, and the general expectation that a greater biomass reduction would be associated with greater shifts in underlying traits. The lack of strong multivariate responses for these traits in this study is likely due to the moderate degree and short‐term application of the drought and the extensive variation among accessions within each treatment and domestication class.

For univariate trait analyses, however, there were some significant drought treatment and treatment by domestication class effects. Drought reduced g_smax_ for cultivated but not wild accessions. This appeared to be due primarily to declining pore length, consistent with reports for some other species (Aasamaa et al., 2001; Bertolino et al., 2019; Doheny‐Adams et al., 2012). Smaller stomata have been argued to provide an advantage because they can open and close more quickly in response to changing plant water status, maximizing CO2 uptake and water‐use efficiency (WUE) when conditions are favorable (Aasamaa et al., 2001; Drake et al., 2013; Hetherington & Woodward, 2003). However, there are still a lot of unknowns about how stomatal density, stomatal size, and g_smax_ relate to drought tolerance in crops (Bertolino et al., 2019; Caine et al., 2019; Hepworth et al., 2015; Hughes et al., 2017).

SLA is an important correlate of plant water use efficiency and drought tolerance (Dong et al., 2020). A lower SLA value, which indicates a higher leaf density and/or leaf thickness, is associated with more efficient water use and better drought tolerance (Onoda et al., 2017; Poorter et al., 2009). In some cases, lower SLA is correlated with higher stomatal density across varying water ability (Xu & Zhou, 2008). In our study, greater plasticity (i.e., greater decline) in SLA in response to drought was associated with increased drought tolerance in both wild and cultivated when analyzed together, making it a promising trait for exploring greater drought tolerance within each domestication class. Although SLA decreased with drought, stomatal density did not decline in repose to drought.

For osmotic adjustment, a previous study found that wild and cultivated *H. annuus* did not differ in their ability to osmotically adjust in response to salinity stress (Tran et al., 2020). This is consistent with our finding in the current study that wild and cultivated *H. annuus* did not differ in their ability to osmotically adjust in response to drought. However, the range in wild accessions was greatly influenced by one accession that had an osmotic adjustment value almost four times larger than the next closest accession, consistent with previous observations of substantive genotypic differences observed within sunflower (Chimenti et al., 2002; Conroy et al., 1988; Rauf & Sadaqat, 2008; Sobrado & Turner, 1983). This suggests that the ability to osmotically adjust may depend on the chosen accessions and that a more extensive survey of wild *H. annuus* populations for ability to osmotically adjust might be useful.

### 4.3 Within domestication classes traits associated with drought tolerance remain elusive

In addition to looking at trait responses to treatment by domestication class, we examined trait variation within each domestication class that might be suggestive of a mechanistic basis of the difference in drought tolerance between cultivated and wild *H. annuus*. In a multivariate approach, the PC axes capturing trait variation in each treatment (control and drought) and the plasticity of traits between treatments were not related to tolerance. This is consistent with not finding a difference between domestication classes in multivariate space and suggests that the traits did not represent a coordinated phenotype associated with accession variation in tolerance.

Additionally, although osmotic adjustment is an oft touted and tested mechanism for drought tolerance, especially in prior work on sunflowers (Chimenti et al., 2002; Conroy et al., 1988; Jones & Turner, 1980; Rauf & Sadaqat, 2008; Sobrado & Turner, 1983), we did not find a relationship between osmotic adjustment and drought tolerance. Due to variability in methodology, genetic and physiological variation of the tested genotypes, and method of enacting drought stress between studies (Blum, 2017; Leport et al., 1999; Serraj & Sinclair, 2002; Turner, 2018; Turner et al., 2007), it is not unprecedented to find no correlation between osmotic adjustment and performance under drought. Our results suggest that the ability of wild *H. annuus* to osmotically adjust is not a promising trait for improving drought tolerance of cultivated *H. annuus*, unless there is variation among wild *H. annuus* populations that has not yet been captured.

However, contrary to osmotic adjustment, the extent of plasticity in SLA was associated with tolerance (Figure 3f). Wild accessions that had a greater reduction in SLA had lower decline in biomass (i.e., greater tolerance). Interestingly, the range and extent of plasticity in SLA were greater in wild than cultivated accessions. This adds to the idea that improving plasticity in certain traits could be a method to improve crop tolerance to stress (Schneider, 2022). However, this association did not differ by domestication class and thus is unlikely to be the basis of the difference between wild and cultivated sunflower for drought tolerance assessed as proportional decline.

Among the traits we measured, g_smax_ plasticity indicates the most potential for contributing to the differences between wild and cultivated for drought tolerance. Wild accessions that had less drought tolerance (i.e., had a larger decline in biomass in response to drought) and had a larger/more consistent decline in g_smax_ than cultivated accessions. The among accession variation in g_smax_ plasticity was weakly associated with drought tolerance and, most importantly, differed by domestication class. Thus, we feel that the contribution of g_smax_ plasticity and its components to drought tolerance of *H. annuus* deserves further investigation.

To further shed light on the impact of anatomical changes to stomatal conductance (i.e., g_smax_), instantaneous operational stomatal conductance should be measured as well since a consistent ratio of measured and theoretical maximum stomatal conductance has not been widely documented (but see Dow et al., 2014; McElwain et al., 2016; Murray et al., 2020). Furthermore, an exploration into the anatomy and biochemistry of stomatal response could also shed light on the factors restricting instantaneous operating stomatal conductance from reaching its maximum potential based on morphology (Rauf & Sadaqat, 2008).

## 5 CONCLUSION

Here, we explored the common expectation that domesticated species have less tolerance to a variety of abiotic stresses, including drought, than their wild progenitors. We tested whether wild and cultivated *H. annuus* differed in drought tolerance assessed as the proportional decline in biomass, when exposed to a moderate drought imposed as minimum soil moisture setpoint that was maintained by frequent small irrigations and plants had less opportunity to avoid low plant water potentials by deeper rooting. Overall, our results support that cultivated *H. annuus* has less tolerance of drought than wild *H. annuus* in this drought scenario. However, there were only weak associations of tolerance with a few traits, including g_smax_ in the drought treatment, SMF in the control treatment, plasticity in SLA, and plasticity g_smax_. Additionally, only the relationship of g_smax_ plasticity to tolerance differed by domestication class, which to our mind makes it the most interesting as a trait potentially contributing to cultivated have less tolerance. Our results highlight the complexities of drought stress and indicate that it may prove difficult to identify single traits that play a large role in variation of tolerance. This reinforces the caution required when considering wild germplasm as a reservoir of unique traits for increased stress tolerance.

## AUTHOR CONTRIBUTIONS

VHT, AAT, and LAD designed the experiment. VHT carried out the experiment and performed initial data analyses. VHT, AAT, and LAD wrote the first manuscript draft. AAT and KMN carried out further data analyses. AAT, KMN, and LAD made subsequent manuscript revisions with all authors approving the manuscript for publication.

## CONFLICT OF INTEREST STATEMENT

The Authors did not report any conflict of interest.

## Supporting information


**Supplemental Figure S1.** Soil moisture content over time.

**Supplemental Figure S2.** Principal component analysis (PCA) of the subset of seven morphological, physiological, and allocational traits plus the six stomatal traits used to calculate g_smax_, for wild (n = 8) and cultivated (n = 8) *Helianthus annuus* in control and drought treatments.

**Supplemental Figure S3.** Principal component analysis (PCA) of g_smax_ (leaf theoretical maximum stomatal conductance) and the six stomatal traits used to calculate it (abaxial stomatal density, adaxial stomatal density, abaxial pore length, adaxial pore length, abaxial guard cell width, and adaxial guard cell width), for wild (n = 8) and cultivated (n = 8) *Helianthus annuus* in control and drought treatments.

**Supplemental Figure S4.** Principal component analyses (PCA) of the of the subset of seven morphological, physiological, and allocational traits under (**a**) Control treatment, (**b**) Drought treatment, and (**c**) Plasticity (the change between treatments). Blue and orange symbols indicate cultivated and wild accessions, respectively.

**Data S1.** Supporting Information.

**Data S2.** Supporting Information.

**Supplemental Table S1.** *Helianthus annuus* accessions used in study, with USDA GRIN (https://www.ars-grin.gov/) identifier and US state where originally collected, if available.
**Supplemental Table S2.** Trait means for each accession and treatment for wild (n = 8) and cultivated (n = 8) *H. annuus* accessions.
**Supplemental Table S3.** Trait loadings on PC axes (companion to Figure 2) Contribution of traits (in %) to variation of the first two principal components of *H. annuus* cultivated and wild accessions under control and drought treatment.
**Supplemental Table S4.** Trait loadings on PC axes (companion to Figure S3). Contribution of traits (in %) to variation of the first two principal components of *H. annuus* cultivated and wild accessions.
