# Supplementary figures and images for: Cultivated sunflower ( Helianthus annuus L.) has lower tolerance of moderate drought stress than its con‐specific wild relative, but the underlying traits remain elusive
Source: Plant Direct. 2024 Apr 4;8(4):e581. doi: 10.1002/pld3.581 (PMC10995449; doi:10.1002/pld3.581)

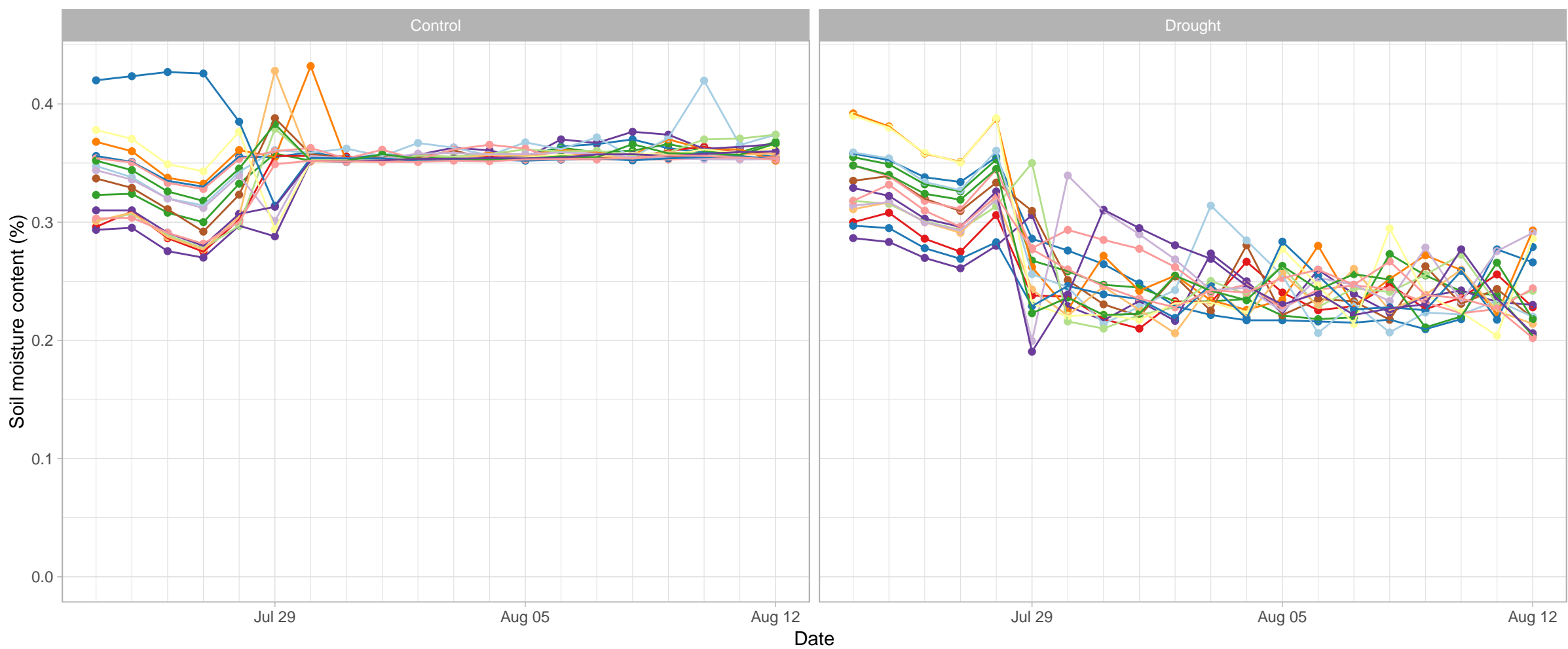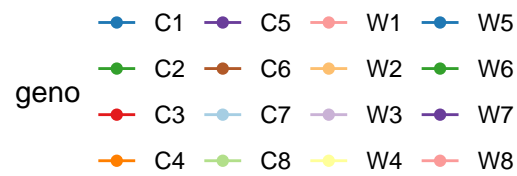

Supplement: Supplementary file 1 — Supplemental Figure S1. Soil moisture content over time. [file PLD3-8-e581-s005.pdf]

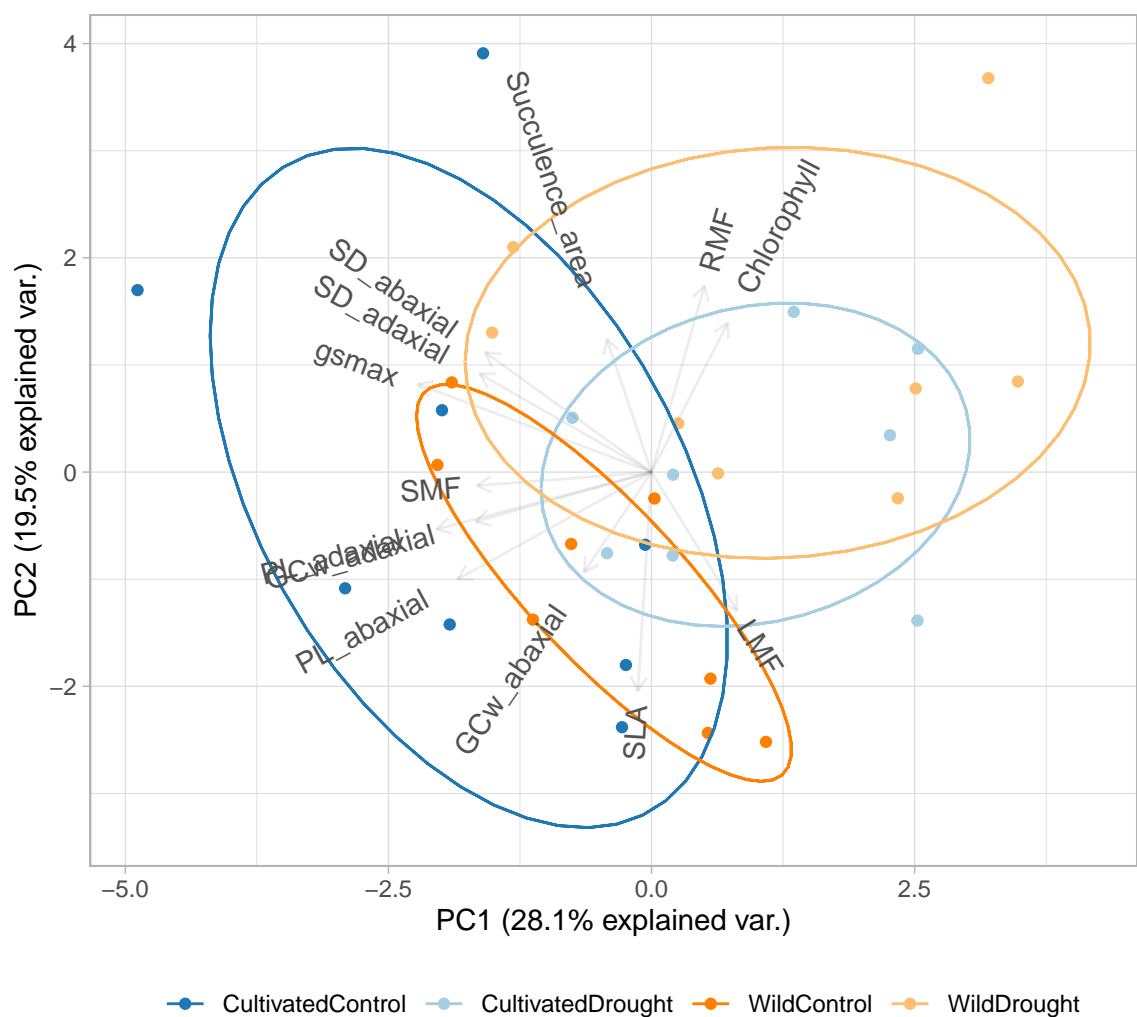

Supplement: Supplementary file 2 — Supplemental Figure S2. Principal component analysis (PCA) of the subset of seven morphological, physiological, and allocational traits plus the six stomatal traits used to calculate gsmax, for wild (n = 8) and cultivated (n = 8) Helianthus annuus in control and drought treatments. [file PLD3-8-e581-s004.pdf]

**(a) Control**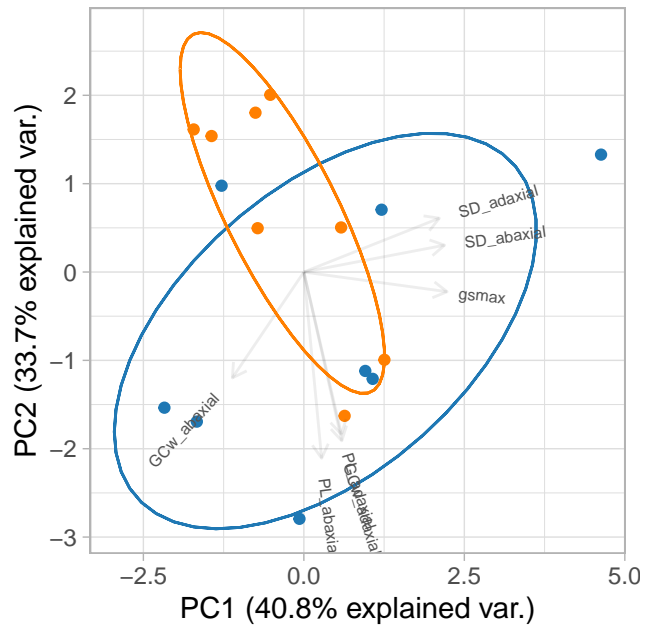**(b) Drought**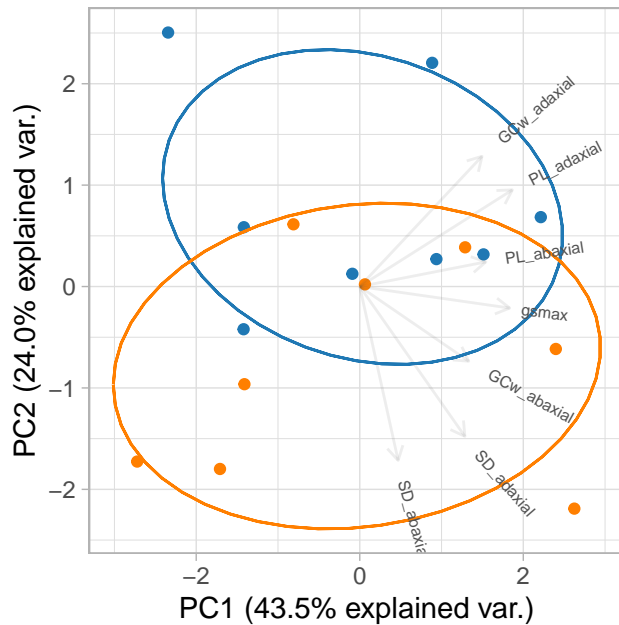**(c) Plasticity**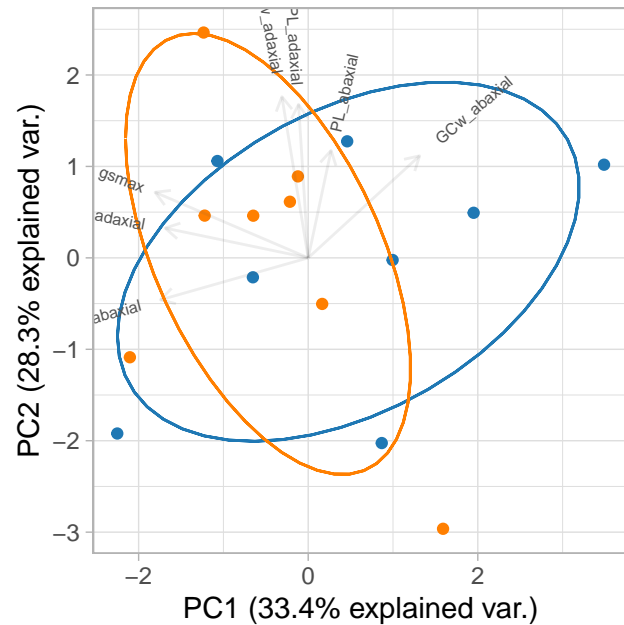

— Cultivated — Wild

Supplement: Supplementary file 3 — Supplemental Figure S3. Principal component analysis (PCA) of gsmax (leaf theoretical maximum stomatal conductance) and the six stomatal traits used to calculate it (abaxial stomatal density, adaxial stomatal density, abaxial pore length, adaxial pore length, abaxial guard cell width, and adaxial guard cell width), for wild (n = 8) and cultivated (n = 8) Helianthus annuus in control and drought treatments. [file PLD3-8-e581-s001.pdf]

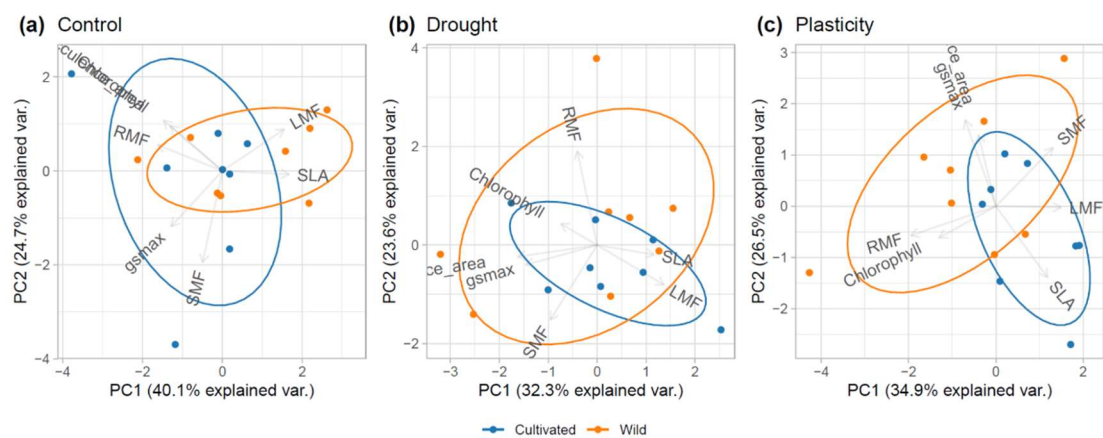

Supplement: Supplementary file 4 — Supplemental Figure S4. Principal component analyses (PCA) of the of the subset of seven morphological, physiological, and allocational traits under (a) Control treatment, (b) Drought treatment, and (c) Plasticity (the change between treatments). Blue and orange symbols indicate cultivated and wild accessions, respectively. [file PLD3-8-e581-s006.pdf]
