# Supplementary material for: Cultivated sunflower ( Helianthus annuus L.) has lower tolerance of moderate drought stress than its con‐specific wild relative, but the underlying traits remain elusive
Source: Plant Direct. 2024 Apr 4;8(4):e581. doi: 10.1002/pld3.581 (PMC10995449; doi:10.1002/pld3.581)
Supplement: Supplementary file 7 — Supplemental Table S1. Helianthus annuus accessions used in study, with USDA GRIN (https://www.ars-grin.gov/) identifier and US state where originally collected, if available. Supplemental Table S2. Trait means for each accession and treatment for wild (n = 8) and cultivated (n = 8) H. annuus accessions. Supplemental Table S3. Trait loadings on PC axes (companion to Figure 2) Contribution of traits (in %) to variation of the first two principal components of H. annuus cultivated and wild accessions under control and drought treatment. Supplemental Table S4. Trait loadings on PC axes (companion to Figure S3). Contribution of traits (in %) to variation of the first two principal components of H. annuus cultivated and wild accessions. [file PLD3-8-e581-s002.docx]

**SUPPLEMENTAL MATERIALS**


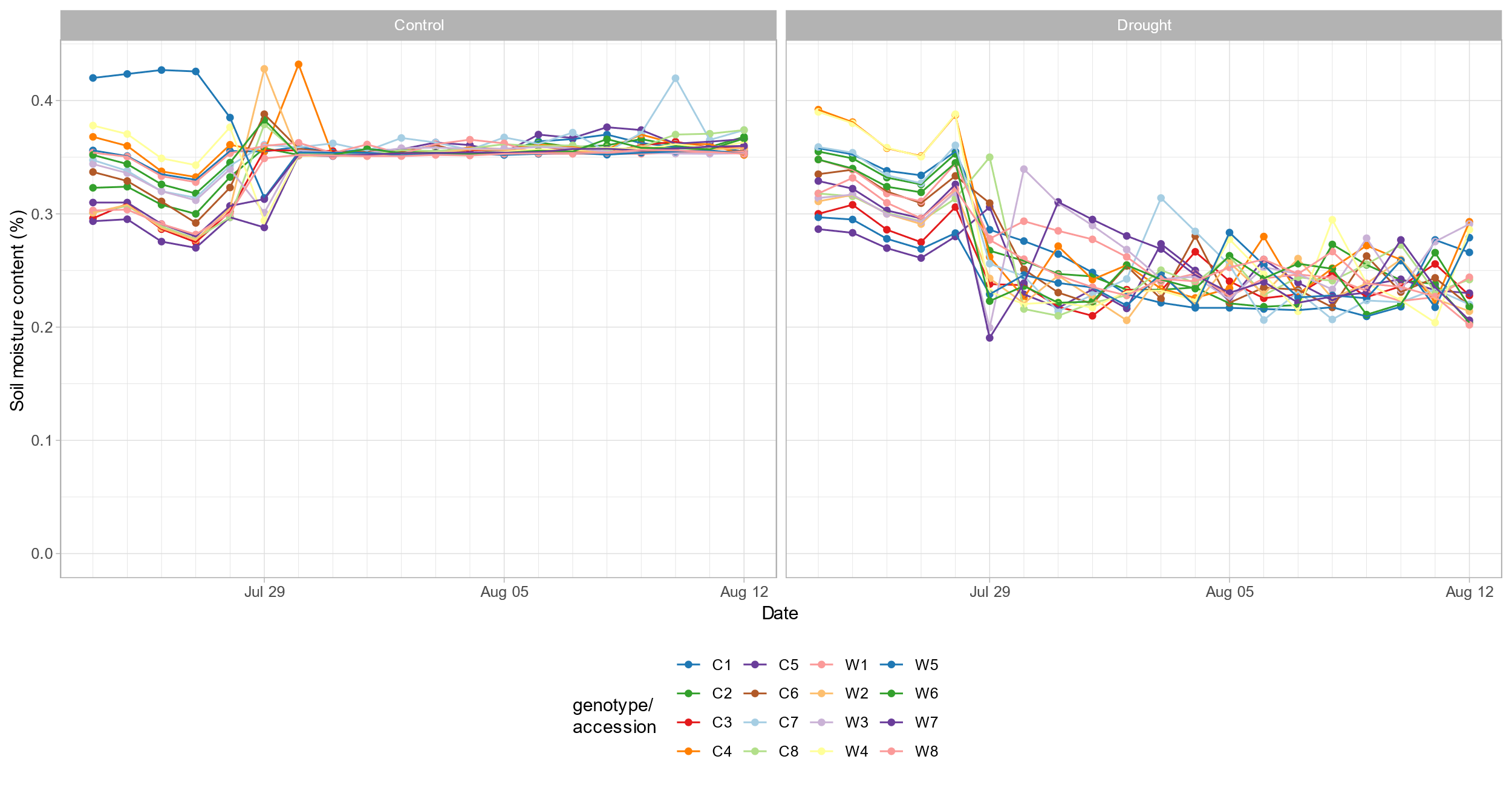


**Supplemental Figure S1.** Soil moisture content over time. For all cultivated (C) genotypes and wild (W) accessions daily soil moisture content in the experiment is shown under control treatment and drought stressed treatment. To reduce visual clutter due to moisture sensor noise, individual points (coloured per genotype/accession) are based on median hourly measurements averaged per individual pot per day. Note the decrease in soil moisture content under drought treatment and the maintenance upon reaching target levels in both treatments.


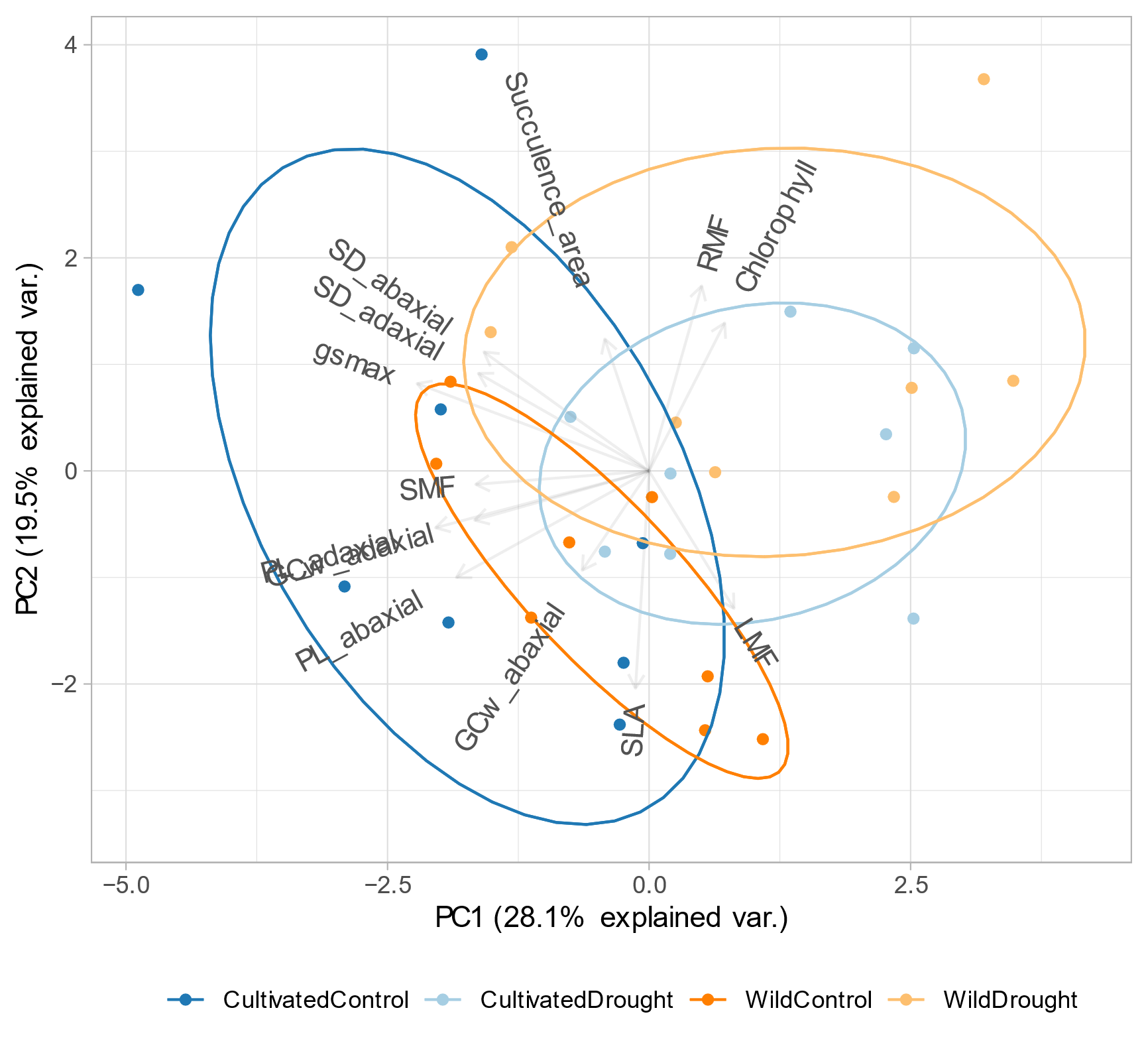


**Supplemental Figure S2**. Principal component analysis (PCA) of the, putatively, size-independent traits plus the six stomatal traits used to calculate g_s max_, for wild (n=8) and cultivated (n=8) *Helianthus annuus* in control and drought treatments. Trait abbreviations: LMF (leaf mass fraction), RMF (root mass fraction), SMF (stem mass fraction), SLA (specific leaf area), succulence, chlorophyll content, and g_s max_ (leaf theoretical maximum stomatal conductance), SD (stomatal density), PL (pore length), GCw (guard cell width, a proxy for pore depth). Blue and orange symbols indicate cultivated and wild accessions, respectively. Darker and lighter hues indicate control and drought treatment, respectively. Hottelings-T^2^ test indicated no significant differences among the four treatment and domestication classes.


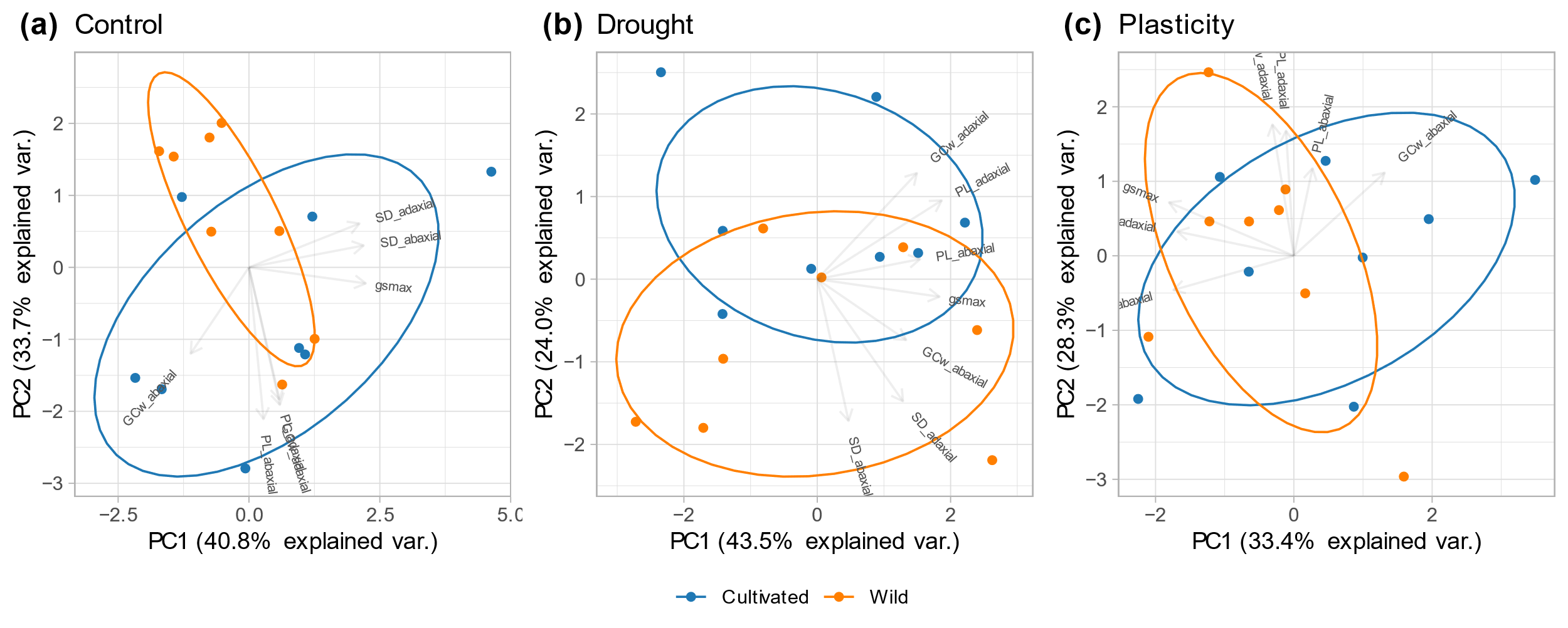


**Supplemental Figure S3**. Principal component analysis (PCA) of g_s max_ (leaf theoretical maximum stomatal conductance) and the six stomatal traits used to calculate it (abaxial stomatal density, adaxial stomatal density, abaxial pore length, adaxial pore length, abaxial guard cell width, and adaxial guard cell width), for wild (n=8) and cultivated (n=8) *Helianthus annuus* in control and drought treatments. Blue and orange symbols indicate cultivated and wild accessions, respectively. The trait loadings for PC1 and 2 are reported in Table S4.


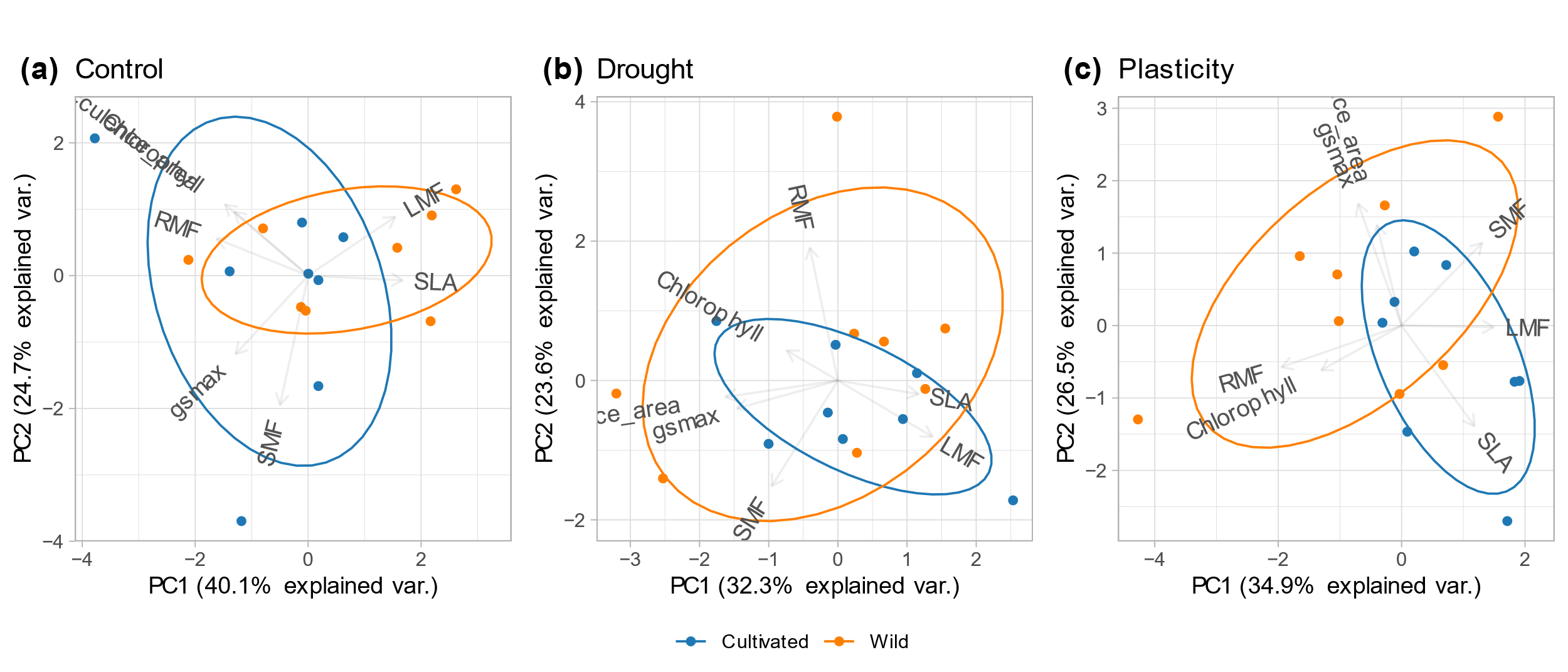


**Supplemental Figure S4.** Principal component analyses (PCA) of the same size-independent traits under (**a**) Control treatment, (**b**) Drought treatment, and (**c**) Plasticity (the change between treatments). Blue and orange symbols indicate cultivated and wild accessions, respectively.

**Supplemental Table S1**. *Helianthus annuus* accessions used in study, with USDA GRIN (<https://www.ars-grin.gov/>) identifier and US state where originally collected, if available.

| Domestication  Status-Accession | USDA GRIN identifier | US state where wild collected |
| --- | --- | --- |
| Cultivated-1 | PI 597373 |  |
| Cultivated-2 | PI 655014 |  |
| Cultivated-3 | PI 64234 |  |
| Cultivated-4 | PI 650817 |  |
| Cultivated-5 | PI 670482 |  |
| Cultivated-6 | Ames 31967 |  |
| Cultivated-7 | PI 599785 |  |
| Cultivated-8 | PI 509053 |  |
| Wild-1 |  | Utah |
| Wild-2 | PI 695784 | Texas |
| Wild-3 | PI 695691 | Utah |
| Wild-4 |  | North Dakota |
| Wild-5 | PI 613729 | Illinois |
| Wild-6 | PI 613752 | Tennessee |
| Wild-7 | PI 494567 | Texas |
| Wild-8 |  | Kansas |

**Supplemental Table S2**. Trait means for each accession and treatment for wild (n=8) and cultivated (n=8) *H. annuus* accessions.
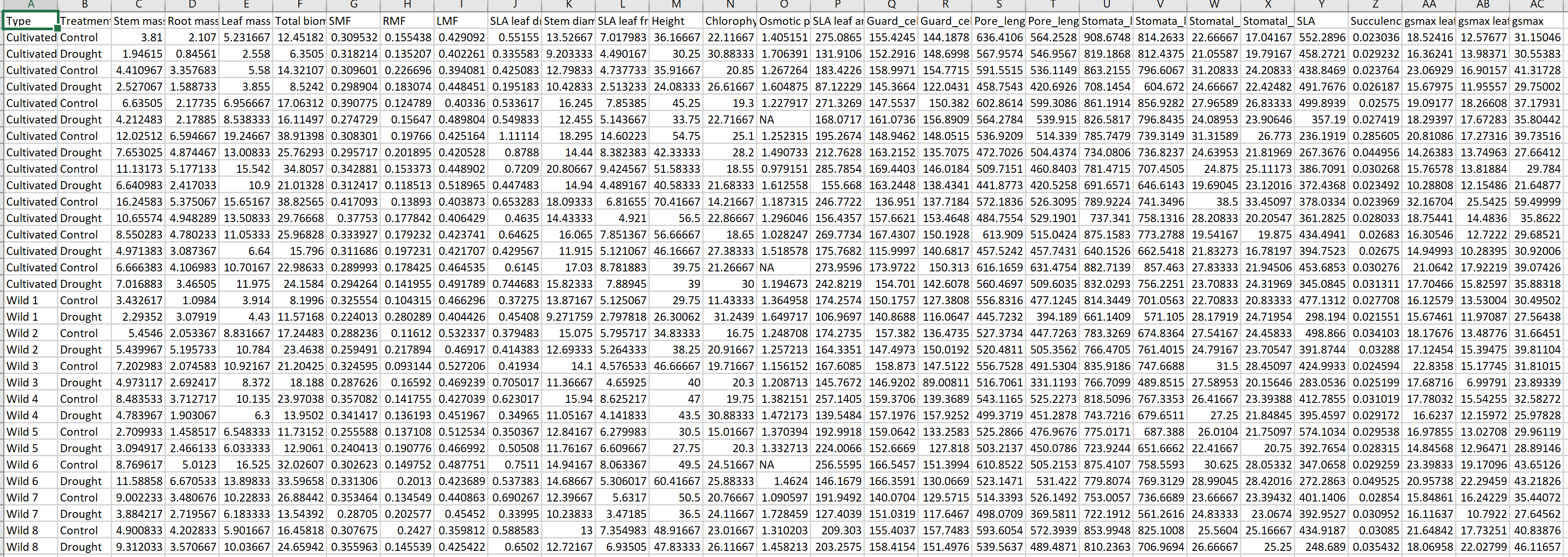


**Supplemental Table S3**. **Trait loadings on PC axes (companion to Figure 2)** Contribution of size independent traits (in %) to variation of the first two principal components of *H. annuus* cultivated and wild accessions under control and drought treatment.


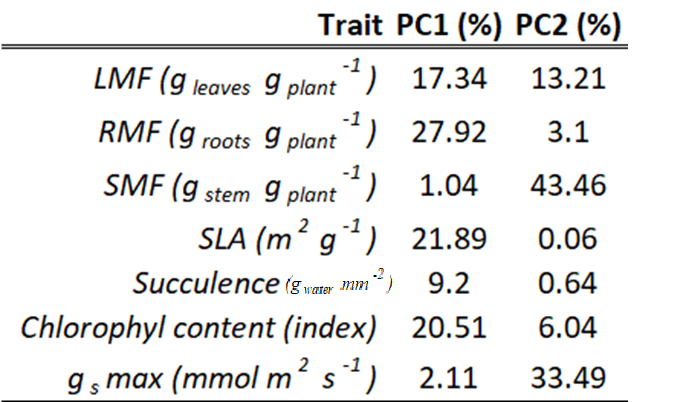


**Supplemental Table S4**. Trait loadings on PC axes (companion to Figure S3). Contribution of traits (in %) to variation of the first two principal components of *H. annuus* cultivated and wild accessions.

|  | Control | | Drought | | Plasticity | |
| --- | --- | --- | --- | --- | --- | --- |
|  | PC1(%) | PC2(%) | PC1(%) | PC2(%) | PC1(%) | PC2(%) |
| *g_s_max (mmol m^2^ s^-1^)* | 30.7 | 0.4 | 22.2 | 0.5 | 29.5 | 5.5 |
| *abaxial stomatal density (mm^-2^)* | 29.6 | 0.7 | 1.5 | 35.3 | 27.5 | 2.2 |
| *adaxial stomatal density (mm^-2^)* | 27.4 | 2.8 | 10.9 | 26.2 | 25.7 | 1.1 |
| *abaxial pore length (µm)* | 0.5 | 33.1 | 15.7 | 0.7 | 0.7 | 14.7 |
| *adaxial pore length (µm)* | 2.1 | 25.1 | 23.1 | 10.9 | 0.1 | 30 |
| *abaxial guard cell width (µm)* | 7.6 | 10.7 | 11.8 | 6.5 | 15.6 | 13.3 |
| *adaxial guard cell width (µm)* | 2.1 | 27.2 | 14.8 | 19.8 | 0.9 | 33.1 |
