# Supplementary material for: Cultivated sunflower ( Helianthus annuus L.) has lower tolerance of moderate drought stress than its con‐specific wild relative, but the underlying traits remain elusive
Source: Plant Direct. 2024 Apr 4;8(4):e581. doi: 10.1002/pld3.581 (PMC10995449; doi:10.1002/pld3.581)
Supplement: Supplementary file 6 — Data S2. Supporting Information. [file PLD3-8-e581-s003.pdf]

Plant Direct
Open Access

Cultivated sunflower (Helianthus annuus L.) has lower tolerance of moderate drought stress than its con-specific wild relative, but the underlying traits remain elusive

Vivian H. Tran1 , Kristen M. Nolting1 , Lisa A. Donovan1,3 * , and Andries A. Temme1,2,3

Decision Letter Round 1:

June 6, 2023

Lisa Donovan
University of Georgia

RE: Cultivated sunflower (Helianthus annuus L.) is less drought tolerant than its con-specific wild relative, but the underlying traits remain elusive.

Dear Dr. Donovan:

Thank you for submitting to Plant Direct. All required reviews have been returned and we have now finished our evaluation of your manuscript. In light of the reviewers' and editor's comments, further revisions are needed before the paper can be accepted for publication in Plant Direct.

Please view the editors' and reviewers' comments below and use their suggestions as a guide while you work on your revision.

When uploading the revised version of this article, please be sure to include the following:

-A word document that contains your response to the reviewers. You should respond to each reviewer comment and note the changes made to the manuscript. If you do not agree with a reviewer's comment and choose not to make a suggested revision, please explain why. Please try to provide as complete an answer as possible to each reviewer's criticisms.
-A tracked changes document with each change highlighted
- A clean version of the latest version of the manuscript

Overall, I find this to be a very interesting and relevant research topic! Please consider revising the manuscript according to the suggestions of the reviewers. I would like to highlight 1) focusing the discussion section a bit more on the results and removing the repetitive parts from the introduction and 2) justifying the use of gsmax as a measure of drought tolerance.

Plant Direct
Open Access

To upload your revision, please click the link below.
https://plantdirect.msubmit.net/cgi-bin/main.plex?el=A6Lr1al6A2ivC2I5A9ftd8LiPHzoOBgd7ugr323pgZ

In order to provide as timely a service as possible, we ask that your revision is resubmitted within three months after receipt of this request. If an extension is needed, please send a request, along with a brief explanation, to the editorial office at plantdirect@wiley.com .

Please note that, in addition to publishing reviewer comments, the author's responses to review comments will also be published alongside the final version of the paper. If you would not like the author's responses to be published, please contact the editorial office at plantdirect@wiley.com .

Thank you very much for giving us an opportunity to review your work. I look forward to receiving the next version.

Sincerely,

Hannah Schneider

Gustavo MacIntosh

Editor, Plant Direct

-- Editor comments:
Overall, I find this to be a very interesting and relevant research topic! Please consider revising the manuscript according to the suggestions of the reviewers. I would like to highlight 1) focusing the discussion section a bit more on the results and removing the repetitive parts from the introduction and 2) justifying the use of gsmax as a measure of drought tolerance.

-- Reviewer comments:
Reviewer #1:

The current manuscript compares cultivated and wild H. annuus accessions in response to soil water deficit. The authors imposed moderate soil drying, using automated irrigation system, and assessed plant biomass and other physiological parameters in H. annuus

accessions. The authors selected 35% of soil moisture as well watered and 20% as drought treatments, which was maintained throughout the experiment. It was acknowledged, in the discussion section, that the imposed soil drying was modest and different scenarios of severe drought can be investigated in the future. Nonetheless, the abstract and the introduction could be tuned down as well. In general, the manuscript is well-written and interesting.
There are a few points I would like to discuss with the authors:
Page 2: Lines 12 - 15: It can be more informative to provide the numbers.
Page 7: Lines 13 - 14: Please add locations of companies.
Page 7: Lines 24 - ...: At which depth was the soil moisture probe placed?
Page 8: Lines 3 - 7: What grantees uniform soil moisture within a pot? Would the authors expect a uniform root length density at this plant age in each pot?
Page 8: Line 5: To complete this, what triggers the closure of the solenoid valves afterward? Was it a fixed period of operating time? What are the uncertainties around 20% and 35%?
Page 8: Line 7: Was it possible to calculate cumulative water consumption for each valve, or operating time?
Page 11: Line 19: Perhaps this result can be introduced in the abstract (24%).

Reviewer #2:

The manuscript of Tran involves contrasting the drought responses of eight genotypes of wild sunflower versus eight genotypes of cultivated sunflower. The work looks in detail at whether the assumption that wild populations have greater drought tolerance than cultivated populations. The paper is well written but fails to adequately answer the question posed at the beginning of the manuscript. The paper is somewhat frustrating and feels like a missed opportunity, the authors could do much more with the dataset at hand.

The authors link theoretical maximum stomatal conductance values to the degree of resistance to drought (measured by the proportional change in biomass). I question the biological value of theoretical gsmax calculations in this type of work given the assumption used in the gsmax formulae that stomata are open. It is like stating the top speed of a car that has run out of gas. Firstly, even under well-watered conditions the predictive capacity of gsmax is poor ($R2 < 0.1$). Under drought stress, the stomata will be closed, so the theoretical gsmax has very little informative value in terms of actual stomatal behaviour. I would recommend performing gas exchange in the future - the Li600 is excellent for this type of work. If you have measured gs data, please include it in the manuscript, as the present data can't support the conclusions drawn within the work.

If you show the values of the individual genotypes, is there a relationship between leaf area and stomatal density (Journal of experimental botany 59, no. 12 (2008): 3317-3325)?

The maintenance of drought levels seems a strength in this work, but by replenishing lost water to maintain a set-point SWC do you produce a buzz-saw effect in plant responses (Journal of Experimental Botany 2015; 66: 2239-2252)?

While some faster growing plants do show a greater proportional response to drought, they also generally maintain higher yields under drought conditions than other varieties that show less proportional decline to water deficit. Can you explore this by comparing different quantification of drought impacts on the varieties?

I appreciate that you provide citations about the 8 accessions of wild and cultivated sunflower (L2-3 P7), but I think the paper would benefit with a greater break down of the different varieties in both the M+M and Results where the accessions seem to be grouped together as wild versus cultivated. This feels like a rather large missed opportunity as there is significant variation in drought resistance in cultivated sunflower varieties and it is strongly correlated to stomatal physiological opening and closing (Physiologia plantarum, 2017, 159(2), 130-147).

Pg 9 - do not cite McElwain et al 2016 here. This paper did not originate the theoretical diffusion method, simply used it following Franks and Farquahar 2001. The correct citation is Cowan 1977

L10-12 of the Discussion is a re-hash of the introduction. There is no need to repeat the basis for your work. You can streamline the paper and make it easier to read by removing unnecessary text.

Plant Direct

Open Access

Decision Letter Round 2:

March 3, 2024
Prof. Lisa Donovan
University of Georgia
Athens, Georgia

MSID: 2023-01186R3
MS TITLE: Cultivated sunflower (Helianthus annuus L.) has lower tolerance of moderate drought stress than its con-specific wild relative, but the underlying traits remain elusive

Dear Dr. Lisa Donovan:

I am pleased to inform you that your manuscript "Cultivated sunflower (Helianthus annuus L.) has lower tolerance of moderate drought stress than its con-specific wild relative, but the underlying traits remain elusive" has been accepted for publication in Plant Direct.

Your article will appear online in the next available issue of Plant Direct. To ensure your article gets published as quickly as possible, please pay attention to the steps detailed below. We have found that most of the delays happen at this stage, especially at the payment stage, so please respond as quickly as possible when prompted.

License Agreement: Once your article has been accepted it will move to Production and undergo admin and file checking - you may receive an email with any queries we have at this stage. When all required items are received by the publisher and queries resolved, the corresponding author will receive an email from Wiley's Author Services system which will ask them to log on at https://authorservices.wiley.com/bauthor and will present them with the appropriate license for completion. Your article cannot be published until both the signed license agreement and payment of the article fee have been received.

Payment of the Open Access Article Publication Fee: All articles published in Plant Direct are fully open access: immediately and freely available to read, download and share. Plant Direct charges a publication fee to cover the publication costs. The corresponding author for this manuscript should have already received a quote from the payments team (cs-openaccess@wiley.com) with the estimated article publication fee; please email cs-openaccess@wiley.com if this has not been received. The corresponding author should log on to the Wiley Author Services site, where the publication fee can be paid by credit card or an invoice. Pro Forma can also be requested. Payment of the publication charge must be received before the article will be published online.

Plant Direct
Open Access

Proofs: You will have the opportunity to look over your paper once more when you receive the author proofs for your article. The proofs will be with you in approximately two weeks. Please note that, in addition to publishing reviewer comments, the author's responses to review comments will also be published alongside the final version of the paper. If you would not like the author's responses to be published, please contact the editorial office at plantdirect@wiley.com .

Promotion of your article: You can help your research get the attention it deserves! Check out Wiley's free Promotion Guide for best-practice recommendations for promoting your work at www.wileyauthors.com/eeo/guide. And learn more about Wiley Editing Services which offers professional video, design, and writing services to create shareable video abstracts, infographics, conference posters, lay summaries, and research news stories for your research at www.wileyauthors.com/eeo/promotion.

Thank you again for your contribution to Plant Direct. If you have any questions, feel free to contact the editorial office at plantdirect@wiley.com .

Sincerely,

Gustavo MacIntosh

Gustavo MacIntosh

Editor, Plant Direct

--- Editor comments

--- Reviewer comments:

Author Response:

Response to Reviewer Comments:

Editor comment: Overall, I find this to be a very interesting and relevant research topic! Please consider revising the manuscript according to the suggestions of the reviewers. I would like to highlight 1) focusing the discussion section a bit more on the results and removing the repetitive parts from the introduction and 2) justifying the use of gsmax as a measure of drought tolerance.

> ***Response:*** *We have revised the manuscript to address the two main concerns you raised and additional detail is provided below.*

Reviewer #1 comment: The current manuscript compares cultivated and wild H. annuus accessions in response to soil water deficit. The authors imposed moderate soil drying, using automated irrigation system, and assessed plant biomass and other physiological parameters in H. annuus accessions. The authors selected 35% of soil moisture as well watered and 20% as drought treatments, which was maintained throughout the experiment. It was acknowledged, in the discussion section, that the imposed soil drying was modest and different scenarios of severe drought can be investigated in the future. Nonetheless, the abstract and the introduction could be tuned down as well.

> ***Response:*** *We have increased the emphasis in the title, abstract and introduction that the drought was moderate*.

Reviewer #1 comment: In general, the manuscript is well-written and interesting.

> ***Response:*** *Thank you.*

Reviewer #1 comment: Page 2: Lines 12 - 15: It can be more informative to provide the numbers.

> ***Response:*** *Our interpretation is that reviewer is requesting the numbers for soil water potentials indicative of drought. However, we were striving for a general opening statement here and the water potential thresholds for responses of different processes and species very greatly. We have revised the text to clarify that we were not referring to specific threshold values for soil water potential.***Reviewer #1 comment:** Page 7: Lines 13 - 14: Please add locations of companies.

> ***Response:*** *The locations for the Scotts Company and Performance Minerals Corporation have been added.*

Reviewer #1 comment: Page 7: Lines 24 - ...: At which depth was the soil moisture probe placed?

__Response:__ The EC 5 probes are each 5 cm long and were inserted so that they were measuring soil from 2-7 cm deep out of approximately 16 cm total depth. This information has been added to the methods.

Reviewer #1 comment: Page 8: Lines 3 - 7: What grantees uniform soil moisture within a pot? Would the authors expect a uniform root length density at this plant age in each pot?

__Response:__ Nemali and van Iersel (2006) has this to say: "When the ϑ measured and maintained by the dielectric moisture sensors was tested using measurements with another probe placed in the same container, the ϑ measured by both probes was found to be similar, indicating that the controller can indeed maintain ϑ near the target level."

However, for all drought methods that we are aware of, it is not reasonable to expect uniform root length density or uniform soil moisture throughout the pot or even the measurement zone. The sensor provides an approximation of the average soil moisture in the sensor zone, and the evidence that a differential water treatment was imposed comes from the significant treatment effect on biomass.

Reviewer #1 comment: Page 8: Line 5: To complete this, what triggers the closure of the solenoid valves afterward? Was it a fixed period of operating time? What are the uncertainties around 20% and 35%?

__Response:__ The solenoid valves were triggered to be open for a fixed period (30 seconds) of irrigation. This information has been added to the methods. The daily averages for soil moisture for each treatment and accession are now presented in supplemental Figure S1.

Reviewer #1 comment: Page 8: Line 7: Was it possible to calculate cumulative water consumption for each valve, or operating time?

__Response:__ Regrettably, it is not possible to collect calculate cumulative water consumption for each pot. The number of times that the irrigation was triggered could be counted, but at this point in the development of our system the valves were not pressure compensated and the pots were not sealed, i.e., water did escape from the bottom of the parts, particularly for the control treatment that approximated field capacity. We relied on the information from the soil moisture probes to trigger the irrigation and characterize our treatments.

Reviewer #1 comment: Page 11: Line 19: Perhaps this result can be introduced in the abstract (24%).

__Response:__ The abstract has been revised to include more detail on the biomass results, including the percentage decline for cultivated accessions.

Reviewer #2 comment: The manuscript of Tran involves contrasting the drought responses of eight genotypes of wild sunflower versus eight genotypes of cultivated sunflower. The work looks in detail at whether the assumption that wild populations have greater drought tolerance than cultivated

populations. The paper is well written but fails to adequately answer the question posed at the beginning of the manuscript.

> ***Response:*** *We are not entirely sure which part of the introduction is being referred to here since specific line numbers were not provided. So, we are basing our response on our interpretation of the sum of reviewer 2's comments and the focus on our study objectives. We feel that we did answer the questions specifically posed at the end of the introduction, at least for the drought conditions imposed in our experiment: we asked whether wild and cultivated H. annuus differ in response to drought for (1) tolerance, (2) individual traits, and (3) trait associations with tolerance. However, as noted by reviewer 1, we do need to avoid the generalization implied by the original title and text because we did not answer the questions posed for all possible combinations of drought severity, duration and implementation. This caveat was initially included in the discussion, and we have revised the manuscript to more prominently acknowledge this caveat throughout.*

Reviewer #2 comment: The paper is somewhat frustrating and feels like a missed opportunity, the authors could do much more with the dataset at hand.

> ***Response****: Based on the sum of comments from reviewer 2, we interpret the "missed opportunity" as referring to the reviewer's preference for more emphasis on 1) comparisons within each domestication class to focus on which specific accessions did "best" in terms of tolerance, and 2) more emphasis on stomatal density and its relationship to other traits. However, our focus was on an empirical test of the assertion in the literature that cultivated plants in general are less tolerant of abiotic stress than wild relatives/progenitors, so we selected cultivated accessions that were genetically diverse and wild accessions that were spatially diverse, as opposed to selecting specific accessions of cultivated and wild based on apriori expectation of degree of drought tolerance. We did look at the association of traits with tolerance* ***within*** *each domestication group (Table 2, Figure 3), and found only a few associations. We have revised the text to clarify these analyses. We feel that an emphasis on specific accessions would be a distraction from our focal questions, but readers with an interest in that will be able to further explore the data using genotype means for individual accessions now included as a supplemental Table S1.*

Reviewer #2 comment: The authors link theoretical maximum stomatal conductance values to the degree of resistance to drought (measured by the proportional change in biomass). I question the biological value of theoretical gsmax calculations in this type of work given the assumption used in the gsmax formulae that stomata are open. It is like stating the top speed of a car that has run out of gas. Firstly, even under well-watered conditions the predictive capacity of gsmax is poor (R2 < 0.1). Under drought stress, the stomata will be closed, so the theoretical gsmax has very little informative value in terms of actual stomatal behaviour. I would recommend performing gas exchange in the future - the Li600 is excellent for this type of work. If you have measured gs data, please include it in the manuscript, as the present data can't support the conclusions drawn within the work.

> ***Response:*** *We appreciate that the capacity of theoretical gsmax to predict instantaneous gs under optimal conditions can be limited, but included it because it has received a lot of*

attention in the literature as an integrated measure of stomatal size and density that can be measured on leaf peels (and thus larger numbers of plants) and is potentially associated with plant performance. Theoretical gsmax did differ for leaves produced under drought and those of comparable leaves for the control plants, and is worth reporting as an integrated measure of acclimation of stomatal density and size that could be associated with tolerance. We did not mean to imply that we expected it to reflect actual gs in the drought treatment, and have modified the text for greater clarity.

We agree that the component traits going in to calculating gsmax are also important to consider and now include them all in Table 1 and give them more attention in the text. Additionally, we now include principle component analysis showing how the component traits relate to gsmax for each treatment and for plasticity (Supplemental Fig S3, Supplemental Table S4), in case a reader is interested.

We prefer to keep the exclusion of the gsmax component traits (stomatal density, pore length, and guard cell width for each surface) from the PCA contrast of the "size-independent" traits in Figure 2, because their inclusion would confound the weighting of gsmax that is calculated from all of those components. However, in case a reader is interested, we now include a PCA of the "size-independent" traits AND the gsmax component traits (Supplemental Fig S3, Supplemental Table S4). Their inclusion did not change the results that for the multivariate analysis (Figure 2 or alternate Figure S2) there were no differences between the treatment by domestication classes.

We agree that instantaneous gas exchange data would also have been valuable, as already stated in the discussion. The instantaneous gs would reflect any short-term effects of water potential on degree of stomatal closure, as well as incorporating any longer-term effects of stomatal pore size and density on gsmax. We had hoped to examine the relationship between calculated gsmax and instantaneous gs for each treatment and domestication class, and whether instantaneous gs differed by domestication class and associated with tolerance. We did attempt leaf gas exchange measurements just prior to harvest using a LiCor 6800. Regrettably, equipment malfunction prevented the collection of usable data so we cannot report instantaneous gas exchange data for this study.

Reviewer #2 comment: If you show the values of the individual genotypes, is there a relationship between leaf area and stomatal density (Journal of experimental botany 59, no. 12 (2008): 3317-3325)?

Response: *We appreciate having the Xu and Zhou 2008 JEB paper brought to our attention. The analysis of leaf trait responses of a native perennial grass (Leymus chinensis) to multiple sustained levels of water stress (ranging from moderate to severe) provides additional context for interpreting the responses of some leaf traits to moderate drought, although not relative drought tolerance. The JEB paper primarily focuses on the negative correlation between*

stomatal density of MRFEL and ***whole canopy SLA*** *across plants at different levels of water stress (their Fig 5), but found no relationship between leaf area per individual plant and stomatal density of MRFEL (see text pg 3320). Xu and Zhou interpret this pattern as suggesting that "a balance of leaf area development with its tissue may be closely linked to the occurrence of guard cells under different water status" (text from page 3320), which suggests a potential tradeoff of higher SLA leaves (larger and/or thinner and/or less dense leaves) having less dense stomata.*

The closest comparable graph that we can generate for our study is a collection of plots of MERFL SLA (i.e., NOT the whole canopy SLA used in Xu and Dodd, but serves as a proxy) by MERFL stomatal density (same trait as in Xu and Zhou) for the 2 levels of soil moisture used in our study. We plotted these data (from Table 1) for cultivated and wild accession in each treatment and leaf surface. Since SLA declined significantly but stomatal density did not (see means contrasts in Table 1), our limited results are not consistent with the potential tradeoff observed in Xu and Zhou 2008.

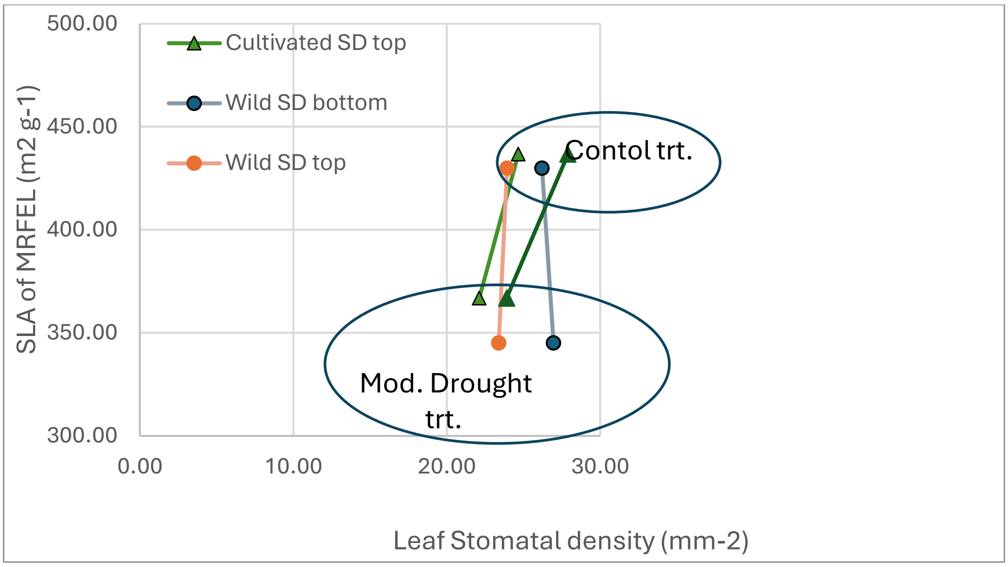

While we now mention Xu and Zhou in the discussion, we decided not include this graph or emphasize this particular bivariate relationship in the revised manuscript for the following reasons: 1) it is not directly related to our objectives, 2) the responses of SLA and stomatal density in response to drought are already included in Table 1, 3) both traits and any correlations (or lack thereof) are already captured by inclusion of gsmax in our multivariate focus characterized in Figure 2 and Figure S4, and 4) a reader interested in focusing on any particular bivariate relationships can now explore this with the accession means now included as supplemental Table S2.

Reviewer #2 comment: The maintenance of drought levels seems a strength in this work, but by replenishing lost water to maintain a set-point SWC do you produce a buzz-saw effect in plant responses (Journal of Experimental Botany 2015; 66: 2239-2252)? ***Response*:** *We appreciate having the Dodd et al. 2015 paper brought to our attention. It does a great job of pointing out the myriad of different ways of experimentally imposing drought stress and that fact that the different methods vary in strengths and weaknesses. We chose a method of repeated soil drying and re-wetting, where the re-wetting in the control treatment was frequent and maintained soil moisture close to field capacity, whereas the re-wetting in the dry treatment was less frequent and maintained a lower average soil moisture level. This is similar to the frequently used method where plants in pots are weighed on a daily basis and water added to the drought treatment to return it to a target soil moisture less than field capacity. But, our automated system allowed the sensing of soil moisture decline and subsequent water addition on a much more frequent basis, and likely resulted in smaller fluctuations of soil moisture than would be experienced with the daily weighing and re-watering method. We feel that the method we used approximated at least one agriculturally relevant scenario (declining soil moisture, then sustained) while avoiding some of the unrealistically fast and extreme swings in soil moisture that can occur in pot studies and introduce an effect of plant size (e.g., bigger plants draw down soil moisture in the pot more quickly and to a lower level before re-wetting). We feel that our choice is one valid drought scenario out of the many that could have been imposed.*

Reviewer #2 comment: While some faster growing plants do show a greater proportional response to drought, they also generally maintain higher yields under drought conditions than other varieties that show less proportional decline to water deficit. Can you explore this by comparing different quantification of drought impacts on the varieties?

Response: *Based on this comment and the next, we interpret this as a request for greater focus on the variation within the domestication classes. However, the focus of our study was to contrast wild and cultivated sunflower for tolerance and associated traits (with accessions as replicates), and not to focus on which particular accession(s) did best within each domestication class, which would best be addressed with a different experimental design. Please see additional comments below.*

Reviewer #2 comment: I appreciate that you provide citations about the 8 accessions of wild and cultivated sunflower (L2-3 P7), but I think the paper would benefit with a greater break down of the different varieties in both the M+M and Results where the accessions seem to be grouped together as wild versus cultivated. This feels like a rather large missed opportunity as there is significant variation in drought resistance in cultivated sunflower varieties and it is strongly correlated to stomatal physiological opening and closing (Physiologia plantarum, 2017, 159(2), 130-147).

Response: *The lack identifiers and sources for the accessions was an oversight on our part and we have added that information as supplemental Table S1. Readers interested in specific accessions can now relate performance and trait data in this study to information found for these accessions in other studies. However, as stated above, we prefer not to add an emphasis*

on which particular accession did "best" because that was not the focus of our study. The Killi et al. 2017 paper cited is indeed interesting, but has a very different focus and setup. It primarily contrasts a C4 (maize) and a C3 species (sunflower), with each species represented by ONE accession that had been assessed apriori as drought tolerant and ONE accession that had been accessed apriori as susceptible. In a growth chamber experiment, a factorial of two temperature treatments (control, high) and watering (well-watered, vs. dry-down and re-wetting to target soil moisture), and plants were followed for trait responses. I don't see any formal quantification of drought tolerance in Killi et al., or any robust tests of trait associations with tolerance (which would require a different experimental design). With all due respect, we focused our paper around the questions stated in our introduction, and found that the wilds were more drought tolerant than cultivated, and that both wild and cultivated traits responded to drought. But, for the traits and responses measured, wilds and cultivated generally did not differ for trait associations with tolerance: only gsmax fit that criteria.

Reviewer #2 comment. The correct citation is Cowan 1977

***Response**: Corrected. Thank you for pointing this out.*

Reviewer #2 comment: L10-12 of the Discussion is a re-hash of the introduction. There is no need to repeat the basis for your work. You can streamline the paper and make it easier to read by removing unnecessary text.

***Response**: The discussion has been revised to address this concern, including removing the initial summary paragraph of the discussion and revising concluding paragrapgh.*
